# Cefepime–taniborbactam activity against antimicrobial-resistant clinical isolates of Enterobacterales and *Pseudomonas aeruginosa*: GEARS global surveillance programme 2018–22

**DOI:** 10.1093/jac/dkae329

**Published:** 2024-09-17

**Authors:** James A Karlowsky, Mark G Wise, Meredith A Hackel, David A Six, Tsuyoshi Uehara, Denis M Daigle, Daniel C Pevear, Greg Moeck, Daniel F Sahm

**Affiliations:** IHMA, Schaumburg, IL, USA; Department of Medical Microbiology and Infectious Diseases, Max Rady College of Medicine, University of Manitoba, Winnipeg, MB, Canada; IHMA, Schaumburg, IL, USA; IHMA, Schaumburg, IL, USA; Venatorx Pharmaceuticals, Inc., Malvern, PA, USA; Venatorx Pharmaceuticals, Inc., Malvern, PA, USA; Venatorx Pharmaceuticals, Inc., Malvern, PA, USA; Venatorx Pharmaceuticals, Inc., Malvern, PA, USA; Venatorx Pharmaceuticals, Inc., Malvern, PA, USA; IHMA, Schaumburg, IL, USA

## Abstract

**Objectives:**

Taniborbactam is a boronate-based β-lactamase inhibitor in clinical development in combination with cefepime.

**Methods:**

Cefepime–taniborbactam and comparator broth microdilution MICs were determined for patient isolates of Enterobacterales (*n *= 20 725) and *Pseudomonas aeruginosa* (*n *= 7919) collected in 59 countries from 2018 to 2022. Taniborbactam was tested at a fixed concentration of 4 mg/L. Isolates with cefepime–taniborbactam MICs** **≥** **16 mg/L underwent WGS. β-Lactamase genes were identified in additional meropenem-resistant isolates by PCR/Sanger sequencing.

**Results:**

Taniborbactam reduced the cefepime MIC_90_ value for all Enterobacterales from >16 to 0.25 mg/L (>64-fold). At ≤16 mg/L, cefepime–taniborbactam inhibited 99.5% of all Enterobacterales isolates; >95% of isolates with MDR and ceftolozane–tazobactam-resistant phenotypes;  ≥ 89% of isolates with meropenem-resistant and difficult-to-treat-resistant (DTR) phenotypes; >80% of isolates with meropenem–vaborbactam-resistant and ceftazidime–avibactam-resistant phenotypes; 100% of KPC-positive, 99% of OXA-48-like-positive, 99% of ESBL-positive, 97% of acquired AmpC-positive, 95% of VIM-positive and 76% of NDM-positive isolates. Against *P. aeruginosa*, taniborbactam reduced the cefepime MIC_90_ value from 32 to 8 mg/L (4-fold). At ≤16 mg/L, cefepime–taniborbactam inhibited 96.5% of all *P. aeruginosa* isolates; 85% of meropenem-resistant phenotype isolates; 80% of isolates with MDR and meropenem–vaborbactam-resistant phenotypes; >70% of isolates with DTR, ceftazidime–avibactam-resistant and ceftolozane–tazobactam-resistant phenotypes; and 82% of VIM-positive isolates. Multiple potential mechanisms of resistance, including carriage of IMP, or alterations in PBP3 (*ftsI*), porins (decreased permeability) and efflux (up-regulation) were present in most isolates with cefepime–taniborbactam MICs** **≥** **16 mg/L.

**Conclusions:**

Cefepime–taniborbactam exhibited potent *in vitro* activity against Enterobacterales and *P. aeruginosa*, and inhibited most carbapenem-resistant isolates, including those carrying serine carbapenemases or NDM/VIM MBLs.

## Introduction

Taniborbactam is a novel bicyclic boronic acid-based, transition state analogue that reversibly and covalently inhibits serine β-lactamases (Ambler class A, C and D β-lactamases), including KPC and OXA-48-like carbapenemases, and that competitively inhibits substrate binding to MBLs (Ambler class B β-lactamases), including NDM, VIM, SPM and GIM, but not IMP.^1–4^ An investigational combination of cefepime, a fourth-generation cephalosporin and taniborbactam has completed Phase 3 clinical development for the treatment of adult patients with complicated urinary tract infection, including acute pyelonephritis.^5^ A second Phase 3 clinical trial is planned to evaluate the efficacy and safety of cefepime–taniborbactam in adults with ventilated hospital-acquired bacterial pneumonia and ventilator-associated bacterial pneumonia (NCT06168734).^6^

The current study assessed the *in vitro* activity of cefepime–taniborbactam and comparators against a global collection of clinical isolates of Enterobacterales and *Pseudomonas aeruginosa* collected from 2018 to 2022 as part of the Global Evaluation of Antimicrobial Resistance via Surveillance (GEARS) programme, characterized the activity of cefepime–taniborbactam against phenotypic and genotypic resistance isolate subsets, and defined resistance mechanisms in isolates with elevated MICs to cephalosporins, carbapenems and cefepime–taniborbactam.

## Materials and methods

### Bacterial isolates

Enterobacterales (*n *= 20 725) and *P. aeruginosa* (*n *= 7919) cultured from patients with community- and hospital-associated infections in 336 sites in 59 countries across five geographic regions (Asia/South Pacific, Europe, Latin America, Middle East/Africa and North America) from 2018 to 2022 were collected and shipped to IHMA (Schaumburg, IL, USA) where their identities (Table S1, available as Supplementary data at *JAC* Online) were confirmed using MALDI-TOF mass spectrometry (Bruker Daltonics, Billerica, MA, USA). Isolates were limited to one per patient per year.

### Antimicrobial susceptibility testing

MICs were determined using the CLSI broth microdilution reference method.^7^ Taniborbactam was supplied by Venatorx Pharmaceuticals, Inc. (Malvern, PA, USA) and tested at a fixed concentration of 4 mg/L with cefepime.^8^ Other agents were purchased from commercial sources. Broth microdilution panels were prepared at IHMA using CAMHB (Becton Dickinson, Sparks, MD, USA) and stored at −70°C until the day of testing. MICs for approved agents were interpreted using 2023 CLSI^8^ and EUCAST^9^ breakpoints. Cefepime–taniborbactam MICs against both Enterobacterales and *P. aeruginosa* were interpreted using provisional breakpoints of ≤16 mg/L (susceptible) and >16 mg/L (resistant).^10^ MDR and difficult-to-treat-resistant (DTR)^11^ isolate definitions are provided in Table S2.

### WGS

Enterobacterales and *P*. *aeruginosa* with cefepime–taniborbactam MIC values of ≥16 mg/L underwent WGS (Tables S2 and S3). Among the 153 of 20 725 (0.7%) Enterobacterales that met this criterion, 47 (30.7%), 45 (29.4%), 33 (21.6%) and 28 (18.3%) isolates, respectively, had cefepime–taniborbactam MICs of 16, 32, 64 and >64 mg/L. Among the 560 of 7919 (7.1%) *P. aeruginosa* that met this criterion, 284 (50.7%), 63 (11.3%), 28 (5.0%), 58 (10.4%) and 127 (22.7%), respectively, had cefepime–taniborbactam MICs of 16, 32, 64, 128 and >128 mg/L. Three *P. aeruginosa* isolates collected in 2021 that tested with cefepime–taniborbactam MIC values** **≥** **16 mg/L were not available for sequencing. Additionally, 30 isolates that originally tested with elevated cefepime–taniborbactam MIC values but upon retesting displayed MIC values** **<** **16 mg/L were subjected to WGS.

### β-Lactamase characterization via PCR and Sanger sequencing

Eight hundred thirty-three Enterobacterales isolates inhibited by cefepime–taniborbactam at ≤8 mg/L and concurrently meropenem-resistant (CLSI criteria)^8^ and 945 randomly selected meropenem non-resistant isolates that concurrently tested with cefepime and/or ceftazidime MIC values** **≥** **2 mg/L were surveyed for their β-lactamase gene content by PCR and Sanger sequencing (Table S2). *P. aeruginosa* isolates with cefepime–taniborbactam MICs of ≤8 mg/L that were meropenem-resistant (CLSI criteria; *n *= 1211)^8^ as well as 100 randomly selected 2018 and 2021 meropenem non-resistant *P. aeruginosa*, testing with ceftazidime and/or cefepime MIC values** **≥** **16 mg/L, also underwent β-lactamase gene PCR and Sanger sequencing.

## Results

### Enterobacterales: phenotype analysis

Among the 20 725 tested isolates of Enterobacterales, the cefepime–taniborbactam MIC_50_ and MIC_90_ were 0.06 and 0.25 mg/L, respectively, and 99.5% of isolates were inhibited by cefepime–taniborbactam at ≤16 mg/L (Table 1). The addition of taniborbactam reduced the cefepime MIC_90_ value by >64-fold (from >16 to 0.25 mg/L).

**Table 1.** *In vitro* activity of cefepime–taniborbactam and comparator agents against 20 725 clinical isolates of Enterobacterales^a^

	MIC, mg/L			MIC interpretation					
				CLSI			EUCAST		
Antimicrobial agent	MIC_50_	MIC_90_	MIC range	% Susceptible	% Intermediate	% Resistant	% Susceptible	% Intermediate	% Resistant
Cefepime–taniborbactam^b^	0.06	0.25	≤0.008 to >64	99.5	NA^d^	0.5	99.5	NA	0.5
Cefepime^c^	≤0.25	>16	≤0.25 to >16	84.2	NA	15.8	76.8	4.4	18.8
Ceftazidime–avibactam	≤0.12	0.5	≤0.12 to >16	97.6	NA	2.4	97.6	NA	2.4
Ceftazidime	0.25	>16	≤0.03 to >16	75.2	2.3	22.5	71.0	4.2	24.8
Ceftolozane–tazobactam	0.5	8	≤0.25 to >8	87.0	2.3	10.7	87.0	NA	13.0
Gentamicin	0.5	>16	≤0.12 to >16	83.7	0.9	15.4	83.7	NA	16.3
Levofloxacin	0.06	>8	≤0.004 to >8	71.6	4.2	24.2	71.6	4.2	24.2
Meropenem–vaborbactam	≤0.06	0.12	≤0.06 to >16	97.3	0.3	2.4	97.6	NA	2.4
Meropenem	0.03	0.12	≤0.004 to >64	94.7	0.6	4.7	95.3	1.1	3.6
Piperacillin–tazobactam	≤4	128	≤4 to >128	80.4	4.1	15.6	80.4	NA	19.6

^a^The 20 725 clinical isolates of Enterobacterales were cultured from patients with community- and hospital-associated infections in 336 sites in 59 countries across five geographic regions (Asia/South Pacific, Europe, Latin America, Middle East/Africa and North America) in the years 2018 (*n *= 4567), 2019 (*n *= 5274), 2020 (*n *= 3886), 2021 (*n *= 4100) and 2022 (*n *= 2898). Patient locations/wards at the time of specimen collection included (*n*/percent of total): general medicine (6985/33.7%); medical ICU (4285/20.4%); general surgery (2505/12.1%); ICU surgery (2003/9.7%); emergency room (1721/8.3%); paediatric ICU (1412/4.7%); general ICU (984/4.8%); general paediatric (669/3.2%); and other/no location given (685/3.3%).

^b^For comparative purposes only, % susceptible and % resistant values for cefepime–taniborbactam correspond to the percentage of isolates inhibited at ≤16 mg/L and ≥32 mg/L, respectively.

^c^For cefepime, susceptible values by CLSI breakpoints include isolates with both susceptible (MIC** **≤** **2 mg/L) and susceptible-dose dependent (MIC 4 and 8 mg/L) MICs.

^d^NA, not applicable.

Cefepime–taniborbactam at ≤16 mg/L inhibited ≥90% of Enterobacterales isolates that were resistant to each of ceftazidime, cefepime, piperacillin–tazobactam, ceftolozane–tazobactam and meropenem; >80% of isolates resistant to each of meropenem–vaborbactam and ceftazidime–avibactam; 96.2% of MDR isolates; and 89.4% of DTR isolates (Table 2). Cefepime–taniborbactam at ≤16 mg/L inhibited a greater percentage of isolates than ceftazidime–avibactam, meropenem–vaborbactam and ceftolozane–tazobactam for each of the nine resistance phenotypes analysed.

**Table 2.** *In vitro* activity of cefepime–taniborbactam and comparator agents against clinical isolates of Enterobacterales with antimicrobial-resistant phenotypes

		MIC, mg/L			MIC interpretation	
Antimicrobial-resistant phenotype, by prevalence (no. of isolates; % of total isolates)^a^	Antimicrobial agent	MIC_50_	MIC_90_	MIC range	CLSI % susceptible	EUCAST % susceptible
Ceftazidime-resistant (4661; 22.5%)	Cefepime–taniborbactam^b^	0.12	2	≤0.008 to >64	97.7	97.7
	Ceftazidime–avibactam	0.5	>16	≤0.12 to >16	89.3	89.3
	Ceftolozane–tazobactam	4	>8	≤0.25 to >8	45.8	45.8
	Meropenem	0.06	32	≤0.004 to >64	77.8	80.0
	Meropenem–vaborbactam	≤0.06	16	≤0.06 to >16	88.2	89.6
	Piperacillin–tazobactam	32	>128	≤4 to >128	34.0	34.0
Cefepime-resistant (3283; 15.8%)	Cefepime–taniborbactam	0.25	4	≤0.008 to >64	96.8	96.8
	Ceftazidime–avibactam	0.5	>16	≤0.12 to >16	85.6	85.6
	Ceftolozane–tazobactam	4	>8	≤0.25 to >8	48.7	48.7
	Meropenem	0.06	>32	≤0.004 to >64	69.8	72.3
	Meropenem–vaborbactam	≤0.06	>16	≤0.06 to >16	83.2	85.1
	Piperacillin–tazobactam	32	>128	≤4 to >128	35.5	35.5
Piperacillin–tazobactam-resistant (3231; 15.6%)	Cefepime–taniborbactam	0.25	4	≤0.008 to >64	96.7	96.7
	Ceftazidime–avibactam	0.5	>16	≤0.12 to >16	85.3	85.3
	Ceftolozane–tazobactam	>8	>8	≤0.25 to >8	26.6	26.6
	Meropenem	0.12	>32	≤0.004 to >64	66.6	70.0
	Meropenem–vaborbactam	≤0.06	>16	≤0.06 to >16	82.5	84.6
	Piperacillin–tazobactam	>128	>128	32 to >128	—	—
MDR phenotype (2730; 13.2%)^c^	Cefepime–taniborbactam	0.25	4	0.015 to >64	96.2	96.2
	Ceftazidime–avibactam	0.5	>16	≤0.12 to >16	82.2	82.2
	Ceftolozane–tazobactam	>8	>8	≤0.25 to >8	33.2	33.2
	Meropenem	0.12	>32	≤0.004 to >64	62.2	65.0
	Meropenem–vaborbactam	≤0.06	>16	≤0.06 to >16	79.4	81.8
	Piperacillin–tazobactam	128	>128	≤4 to >128	20.3	20.3
Ceftolozane–tazobactam-resistant (2218; 10.7%)	Cefepime–taniborbactam	0.5	8	0.015 to >64	95.4	95.4
	Ceftazidime–avibactam	1	>16	≤0.12 to >16	77.6	77.6
	Ceftolozane–tazobactam	>8	>8	8 to >8	—	—
	Meropenem	1	64	≤0.004 to >64	52.9	57.4
	Meropenem–vaborbactam	0.12	>16	≤0.06 to >16	74.9	77.9
	Piperacillin–tazobactam	>128	>128	≤4 to >128	4.7	4.7
Meropenem-resistant (972; 4.7%)	Cefepime–taniborbactam	1	32	0.015 to >64	89.6	89.6
	Ceftazidime–avibactam	4	>16	≤0.12 to >16	54.4	54.4
	Ceftolozane–tazobactam	>8	>8	1 to >8	1.2	1.2
	Meropenem	32	>64	4 to >64	—	—
	Meropenem–vaborbactam	16	>16	≤0.06 to >16	41.9	48.8
	Piperacillin–tazobactam	>128	>128	≤4 to >128	0.3	0.3
DTR phenotype (939; 4.5%)^d^	Cefepime–taniborbactam	2	32	0.015 to >64	89.4	89.4
	Ceftazidime–avibactam	4	>16	≤0.12 to >16	56.1	56.1
	Ceftolozane–tazobactam	>8	>8	2 to >8	0.2	0.2
	Meropenem	32	>64	2 to >64	0	7.9
	Meropenem–vaborbactam	8	>16	≤0.06 to >16	44.7	51.0
	Piperacillin–tazobactam	>128	>128	32 to >128	0	0
Ceftazidime–avibactam-resistant (499; 2.4%)	Cefepime–taniborbactam	2	64	0.03 to >64	81.4	81.4
	Ceftazidime–avibactam	>16	>16	16 to >16	—	—
	Ceftolozane–tazobactam	>8	>8	≤0.25 to >8	0.2	0.2
	Meropenem	32	>64	≤0.004 to >64	8.4	11.2
	Meropenem–vaborbactam	>16	>16	≤0.06 to >16	20.8	28.3
	Piperacillin–tazobactam	>128	>128	≤4 to >128	3.8	3.8
Meropenem–vaborbactam-resistant (498; 2.4%)	Cefepime–taniborbactam	2	64	0.12 to >64	81.5	81.5
	Ceftazidime–avibactam	>16	>16	≤0.12 to >16	28.1	28.1
	Ceftolozane–tazobactam	>8	>8	1 to >8	1.0	1.0
	Meropenem	>32	>64	4 to >64	0	0
	Meropenem–vaborbactam	>16	>16	16 to >16	—	—
	Piperacillin–tazobactam	>128	>128	8 to >128	0.2	0.2

^a^Resistant phenotypes determined using CLSI breakpoints. % of total isolates calculated as % of 20 725 clinical isolates of Enterobacterales.

^b^For comparative purposes only, % susceptible values for cefepime–taniborbactam correspond to the percentage of isolates inhibited at ≤16 mg/L.

^c^An MDR phenotype was assigned to isolates resistant, using 2023 CLSI breakpoints,^8^ to at least one agent from ≥3 of the following antimicrobial agent classes: aminoglycosides (gentamicin), β-lactam combination agents (piperacillin–tazobactam, ceftazidime–avibactam, ceftolozane–tazobactam, meropenem–vaborbactam), carbapenems (meropenem or imipenem), cephems (ceftazidime, cefepime) and fluoroquinolones (levofloxacin or ciprofloxacin).

^d^DTR isolates were identified using the definition of Kadri *et al*.^11^ as isolates intermediate or resistant, by 2023 CLSI breakpoints,^8^ to fluoroquinolones (levofloxacin) and all β-lactams including carbapenems and piperacillin–tazobactam, but excluding ceftazidime–avibactam, ceftolozane–tazobactam and meropenem–vaborbactam.

### Enterobacterales: genotype analysis

In total, 1931 Enterobacterales isolates were subjected to molecular testing by WGS or PCR and Sanger sequencing. Cefepime–taniborbactam (MIC_50_, 1 mg/L; MIC_90_, 16 mg/L), ceftazidime–avibactam (MIC_50_, 4 mg/L; MIC_90_, >16 mg/L) and meropenem–vaborbactam (MIC_50_, 16 mg/L; MIC_90_, >16 mg/L) inhibited 90.1%, 54.6% and 42.7%, respectively, of carbapenemase-positive isolates of Enterobacterales. Cefepime–taniborbactam inhibited 77.5% of MBL-positive isolates overall, including 95.2% of VIM-positive and 75.5% of NDM-positive isolates. Cumulative MIC distributions stratified by carbapenemase type for the 961 IMP-negative isolates of carbapenemase-positive Enterobacterales (that carried a single carbapenemase) are shown in Table S4. The most active comparator, meropenem–vaborbactam, inhibited only 8.5% of MBL-positive Enterobacterales isolates.

Cefepime–taniborbactam (100% inhibited at ≤16 mg/L), ceftazidime–avibactam (94.8% susceptible) and meropenem–vaborbactam (92.7% susceptible) were active against Enterobacterales carrying KPCs (Table 3). Cefepime–taniborbactam (98.7% inhibited at ≤16 mg/L) and ceftazidime–avibactam (95.2% susceptible) were also highly active against Enterobacterales carrying OXA-48-like enzymes, while meropenem–vaborbactam was not (32.9% susceptible). Cefepime–taniborbactam (at ≤16 mg/L), ceftazidime–avibactam, meropenem–vaborbactam and meropenem inhibited ≥90% of ESBL-positive and acquired AmpC-positive isolates.

**Table 3.** *In vitro* activity of cefepime–taniborbactam and comparator agents against 1931 clinical isolates of Enterobacterales with molecularly identified β-lactamase genotypes

		MIC, mg/L			MIC interpretation	
Genotype (no. of isolates; % of total molecularly characterized isolates)	Antimicrobial agent	MIC_50_	MIC_90_	MIC range	CLSI % susceptible	EUCAST % susceptible
Carbapenemase-positive (968; 50.1%)^a^	Cefepime–taniborbactam^b^	1	16	0.015 to >64	90.1	90.1
	Ceftazidime–avibactam	4	>16	≤0.12 to >16	54.6	54.6
	Ceftolozane–tazobactam	>8	>8	0.5 to >8	1.2	1.2
	Meropenem	32	>64	0.03 to >64	4.5	6.9
	Meropenem–vaborbactam	16	>16	≤0.06 to >16	42.7	49.2
	Piperacillin–tazobactam	>128	>128	≤4 to >128	0.4	0.4
MBL-positive (413; 21.4%)^c^	Cefepime–taniborbactam	2	64	0.03 to >64	77.5	77.5
	Ceftazidime–avibactam	>16	>16	0.25 to >16	0.5	0.5
	Ceftolozane–tazobactam	>8	>8	>8 to >8	0	0
	Meropenem	32	>64	0.5 to >64	1.9	2.7
	Meropenem–vaborbactam	>16	>16	0.25 to >16	8.5	17.2
	Piperacillin–tazobactam	>128	>128	≤4 to >128	0.7	0.7
NDM-positive (364; 18.9%)^d^	Cefepime–taniborbactam	2	64	0.06 to >64	75.5	75.5
	Ceftazidime–avibactam	>16	>16	0.25 to >16	0.3	0.3
	Ceftolozane–tazobactam	>8	>8	>8 to >8	0	0
	Meropenem	>32	>64	2 to >64	0	0.3
	Meropenem–vaborbactam	>16	>16	2 to >16	3.3	11.5
	Piperacillin–tazobactam	>128	>128	32 to >128	0.0	0.0
VIM-positive (42; 2.2%)^d^	Cefepime–taniborbactam	1	8	0.03 to 32	95.2	95.2
	Ceftazidime–avibactam	>16	>16	4 to >16	2.4	2.4
	Ceftolozane–tazobactam	>8	>8	>8 to >8	0	0
	Meropenem	8	64	0.5 to >64	7.1	11.9
	Meropenem–vaborbactam	8	>16	0.5 to >16	42.9	52.4
	Piperacillin–tazobactam	>128	>128	32 to >128	0	0
KPC-positive (327; 16.9%)^e^	Cefepime–taniborbactam	0.5	4	0.015 to 8	100	100
	Ceftazidime–avibactam	2	8	≤0.12 to >16	94.8	94.8
	Ceftolozane–tazobactam	>8	>8	0.5 to >8	1.2	1.2
	Meropenem	32	>64	0.03 to >64	2.8	4.6
	Meropenem–vaborbactam	0.12	4	≤0.06 to >16	92.7	96.6
	Piperacillin–tazobactam	>128	>128	≤4 to >128	0.3	0.3
OXA-48 group-positive (228; 11.8%)^f^	Cefepime–taniborbactam	2	4	0.03 to >64	98.7	98.7
	Ceftazidime–avibactam	1	4	≤0.12 to >16	95.2	95.2
	Ceftolozane–tazobactam	>8	>8	1 to >8	3.5	3.5
	Meropenem	16	64	0.12 to >64	11.8	18.0
	Meropenem–vaborbactam	16	>16	0.12 to >16	32.9	39.0
	Piperacillin–tazobactam	>128	>128	64 to >128	0	0
ESBL-positive (794; 41.1%)^g^	Cefepime–taniborbactam	0.12	1	0.015 to 64	99.1	99.1
	Ceftazidime–avibactam	0.25	1	≤0.12 to >16	98.5	98.5
	Ceftolozane–tazobactam	1	>8	≤0.25 to >8	75.1	75.1
	Meropenem	0.03	0.12	≤0.004 to 64	93.3	94.8
	Meropenem–vaborbactam	≤0.06	0.12	≤0.06 to >16	99.4	99.6
	Piperacillin–tazobactam	8	>128	≤4 to >128	57.8	57.8
Acquired AmpC-positive (66; 3.4%)^h^	Cefepime–taniborbactam	0.06	2	0.015 to 32	97.0	97.0
	Ceftazidime–avibactam	0.25	2	≤0.12 to >16	97.0	97.0
	Ceftolozane–tazobactam	2	>8	≤0.25 to >8	60.6	60.6
	Meropenem	0.06	0.25	0.015 to 8	93.9	95.5
	Meropenem–vaborbactam	≤0.06	0.12	≤0.06 to 4	100	100
	Piperacillin–tazobactam	8	>128	≤4 to >128	60.6	60.6

^a^Includes isolates with MBLs and serine carbapenemases; isolates could also possess OSBLs (original spectrum β-lactamases, e.g. TEM-1 and SHV-1), ESBLs or AmpC-type enzymes.

^b^For comparative purposes only, % susceptible values for cefepime–taniborbactam correspond to the percentage of isolates inhibited at ≤16 mg/L.

^c^Includes isolates that possess IMP alone (*n *= 7), NDM alone (*n *= 363), VIM alone (*n *= 42) and NDM + IMP (*n *= 1).

^d^Isolates could also possess serine carbapenemases, ESBLs, AmpCs and/or OSBLs, but no other MBLs.

^e^Isolates could also possess OXA-48 group, ESBLs, AmpC-type enzymes and OSBLs but not MBLs.

^f^Isolates could also possess ESBLs, AmpC-type enzymes or OSBLs, but no other carbapenemases.

^g^Isolates could also possess AmpC-type enzymes, or OSBLs, but no carbapenemases.

^h^Isolates could also possess OSBLs, but no ESBLs or carbapenemases.

**Figure 1.**
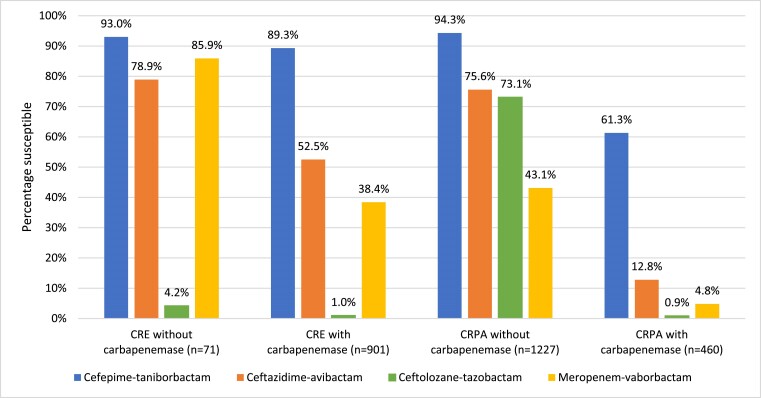
*In vitro* activity of cefepime–taniborbactam^a^ and comparator agents^b^ against carbapenem-resistant^c^ clinical isolates of Enterobacterales (CRE) and *P. aeruginosa* (CRPA) stratified by the presence and absence of molecularly identified carbapenemases. ^a^For comparative purposes only, % susceptible values for cefepime–taniborbactam correspond to the percentage of isolates inhibited at ≤16 mg/L. ^b^CLSI and EUCAST susceptible breakpoints for ceftazidime–avibactam and ceftolozane–tazobactam are harmonized. As CLSI does not publish breakpoints for meropenem–vaborbactam against *P. aeruginosa*, the EUCAST susceptible breakpoint is used. ^c^Carbapenem-resistant phenotypes for Enterobacterales and *P. aeruginosa* were based on meropenem susceptibility using CLSI 2023 breakpoints. This figure appears in colour in the online version of *JAC* and in black and white in the print version of *JAC*.

At least 92.7% (901/972) of meropenem-resistant Enterobacterales isolates carried one or more carbapenemase genes detectable by the methods used (Figure 1). Against the 972 meropenem-resistant Enterobacterales isolates identified in the study, cefepime–taniborbactam inhibited 37%–88% more isolates with carbapenemases (*n *= 901) and 7%–89% more isolates without carbapenemases (*n *= 71) than the other newer β-lactam/β-lactamase inhibitor combinations tested. Overall, 37.1% (334/901) and 21.1% (15/71) of meropenem-resistant Enterobacterales isolates with and without carbapenemases had a cefepime–taniborbactam-susceptible/ceftazidime–avibactam-resistant phenotype, respectively. Less than 80% (56/71) of meropenem-resistant Enterobacterales isolates without carbapenemases were ceftazidime–avibactam-susceptible compared to 93% (66/71) for cefepime–taniborbactam.

### Enterobacterales: characterization of isolates with elevated cefepime–taniborbactam MICs

The 153 of 20 725 Enterobacterales isolates with cefepime–taniborbactam MICs** **≥** **16 mg/L [47 (30.7%) of which had MIC of 16 mg/L] consisted of 93 *Klebsiella pneumoniae*, 36 *Escherichia coli*, 10 *Providencia rettgeri*, three *Providencia stuartii*, three *Serratia marcescens*, three *Enterobacter cloacae*, two *Enterobacter hormaechei*, two *Citrobacter freundii* and one *Morganella morganii*. Every isolate carried one or more β-lactamase genes; 89.6% of isolates carried one or more carbapenemase genes. Nearly all (104/106; 98%) Enterobacterales with cefepime–taniborbactam MICs** **>** **16 mg/L were MDR, and only 10.6%, 7.7% and 0% of these isolates were susceptible to ceftazidime–avibactam, meropenem–vaborbactam and ceftolozane–tazobactam, respectively.

Putative explanations for elevated cefepime–taniborbactam MICs could be deduced for 91 of 93 *K. pneumoniae* isolates. Two isolates carried IMP-8. Eighty of the remaining 91 isolates harboured NDM in combination with other resistance factors like porin mutations that may result in decreased susceptibility. One isolate had a *ftsI* gene (PBP3; the primary target of cefepime) encoding a four amino acid insertion (TVPY) at position 334 and also possessed NDM-5, OXA-181 (an OXA-48-group carbapenemase) and CTX-M-15 (each within spectrum of taniborbactam). Eighty-nine of the 93 isolates (95.7%) had a porin disruption or alteration likely affecting drug entry through the outer membrane, including 76 isolates exhibiting a disruption in *ompK35* (OmpF homologue) and 89 exhibiting a disruption or insertion in *ompK36* (OmpC homologue) in regions of each porin known to affect permeability. Overall, 81.7% (76/93) of isolates showed disruptions or relevant insertions in both major porins. Three isolates showed a disruption in *ramR*, and for four isolates the gene coding for this protein appeared to be deleted. RamR, a negative regulator of RamA, is an enhancer of AcrAB efflux pump expression (and multidrug resistance) in *K. pneumoniae*.^12^

Putative factors contributing to elevated cefepime–taniborbactam MICs were identified in 34 of 36 (94.4%) *E. coli* isolates. Thirty-one of the 36 (86.1%) isolates (14/31 with cefepime–taniborbactam MIC of 16 mg/L and 17/31 with cefepime–taniborbactam MIC** **>** **16 mg/L) showed four amino acid insertions (YRIN or YRIK) at position 333 in PBP3 known to negatively impact accessibility of cephalosporins and aztreonam to the transpeptidase binding site.^13^ Of the five remaining isolates, one had a novel four amino acid insertion ‘IPYR’ at amino acid position 331, one had a single substitution (I332V) and three exhibited wild-type *ftsI*. Twenty-eight isolates carried NDM. All but one of the NDM-positive isolates also possessed one of the aforementioned PBP3 insertions. Previous testing demonstrated that some *E. coli* isolates possessing these PBP3 insertions were inhibited by cefepime–taniborbactam at ≤8 mg/L, suggesting that these insertions alone should not be considered the unique basis for resistance.^10^ Thirty-two of the 36 isolates (88.9%) had an alteration in OmpC and/or OmpF likely resulting in reduced permeability. Genetic lesions suggestive of a non-functional protein were observed for *marR* (AcrAB efflux pump regulatory gene) in one isolate, *marA* in one isolate and *acrR* (another AcrAB efflux pump regulatory gene) in 20 other isolates. Table 4 summarizes the overlap of mutations in porin genes, *ftsI* and efflux regulatory genes in *E. coli* isolates with elevated cefepime–taniborbactam MICs. Nine of the 16 *E. coli* with cefepime–taniborbactam MICs of 16 mg/L and nine of the 20 with cefepime–taniborbactam MICs of >16 mg/L exhibited all three of the putative resistance mechanisms: porin disruption, PBP3 sequence variation and efflux up-regulation.

**Table 4.** Occurrence and co-occurrence of putative non-β-lactamase resistance mechanisms identified in 36 isolates of *E. coli* with cefepime–taniborbactam MICs** **≥** **16 mg/L

	Cefepime–taniborbactam MIC (number of isolates)	
	16 mg/L (*n *= 16)	>16 mg/L (*n *= 20)
Putative resistance mechanism(s)^a^	Number of isolates (%)	Number of isolates (%)
Porin disruption and PBP3 sequence variation and efflux up-regulation	9 (56.2)	9 (45.0)
Porin disruption and PBP3 sequence variation	4 (25.0)	9 (45.0)
Porin disruption alone	0	1 (5.0)
PBP3 sequence variation alone	0	0
PBP3 sequence variation and efflux up-regulation	1 (6.2)	0
Efflux up-regulation	1 (6.2)	0
No putative resistance mechanism(s) identified	1 (6.2)	1 (5.0)

^a^Major porin genes, *ompC* and *ompF*, were screened for alterations that code for a truncated, presumably non-functional protein; PBP3 sequence variation included *ftsI* sequences predicted to code for insertions known to reduce cefepime binding;^13^ and efflux up-regulation included genetic changes that likely enhance drug extrusion via AcrAB-TolC efflux pump.^14^

Among the remaining 24 Enterobacterales isolates with elevated cefepime–taniborbactam MICs, one *C. freundii* isolate carried an IMP-8 gene and one displayed a gross disruption in OmpF porin. Two of the *E. cloacae* isolates carried an IMP enzyme (IMP-4 and IMP-8). For the third isolate of *E. cloacae*, no clear explanation for the elevated cefepime–taniborbactam MIC was found, although the deduced PBP3 amino acid sequence showed several substitutions when compared to the reference sequence. One *E. hormaechei* isolate carried IMP-8, and the second isolate exhibited a ‘YRIN’ insertion in PBP3. Among the 10 *P. rettgeri* isolates (nine of which had a cefepime–taniborbactam MIC of ≥64 mg/L), one harboured IMP-27 and eight others NDM-1. All *P. rettgeri* carried at least one acquired OXA-type enzyme, and five carried a VEB-type ESBL (taniborbactam is expected to inhibit both OXA and VEB β-lactamases in addition to NDM-1).^2^ Two isolates of *P. rettgeri* showed a disruption in the gene coding for its major porin, an OmpF homologue. Unidentified concurrent resistance mechanisms must have contributed to the elevated cefepime–taniborbactam MICs in the 10 *P. rettgeri* isolates. The three *S. marcescens* isolates carried CTX-M-15 and OXA-1. Reduced susceptibility to cefepime has been linked to OXA-1 carriage in Enterobacterales.^15^

### Enterobacterales: regional, specimen source and species analyses

**Figure 2.**
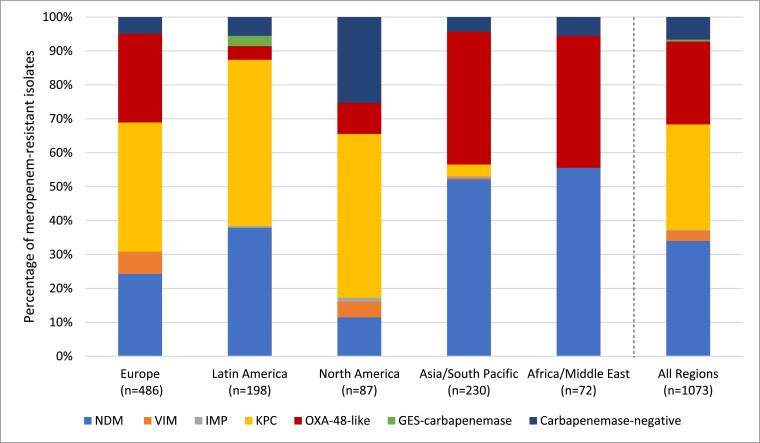
Global region diversity of carbapenemases detected among meropenem-resistant Enterobacterales^a^. ^a^Isolates that carried multiple carbapenemases were counted for each carbapenemase type. This figure appears in colour in the online version of *JAC* and in black and white in the print version of *JAC*.

Meropenem susceptible values across the five global regions studied ranged from 98%–99% in North America to 91%–92% in Latin America (Table 5). Cefepime–taniborbactam at ≤16 mg/L inhibited from 98.0% (Asia/South Pacific) to 99.9% (North America) of Enterobacterales across the five global regions. NDM was the predominant carbapenemase among meropenem-resistant Enterobacterales (defined by CLSI criteria) in Asia/South Pacific and Africa/Middle East regions, while KPC was most frequent in Europe, Latin America and North America (Figure 2). Across five common specimen sources, nearly all isolates (99.3%–99.8%) had cefepime–taniborbactam MICs of ≤16 mg/L (Table S5). Cefepime–taniborbactam MIC_90_ values differed by up to 16-fold across the Enterobacterales species tested (Table S6).

**Table 5.** *In vitro* activity of cefepime–taniborbactam, cefepime and meropenem against 20 725 clinical isolates of Enterobacterales stratified by global region of collection

		MIC, mg/L			MIC interpretation	
	Antimicrobial agent	MIC_50_	MIC_90_	MIC range	CLSI % susceptible	EUCAST % susceptible
Region of collection (no. of isolates; % of total isolates)						
Asia/South Pacific (2467; 11.9%)	Cefepime–taniborbactam^a^	0.06	0.5	≤0.25 to >16	98.0	98.0
	Cefepime^b^	≤0.25	>16	≤0.008 to >64	79.4	71.2
	Meropenem	0.03	0.25	≤0.004 to >64	92.1	92.5
Europe (8760; 42.3%)	Cefepime–taniborbactam	0.06	0.25	≤0.25 to >16	99.6	99.6
	Cefepime	≤0.25	>16	≤0.008 to 64	83.9	77.3
	Meropenem	0.03	0.12	≤0.004 to >64	94.1	94.8
Latin America (2331; 11.2%)	Cefepime–taniborbactam	0.06	0.5	≤0.25 to >16	99.7	99.7
	Cefepime	≤0.25	>16	≤0.008 to 64	75.9	66.2
	Meropenem	0.03	0.5	≤0.004 to >64	91.3	92.2
Middle East/Africa (1332; 6.4%)	Cefepime–taniborbactam	0.06	0.5	≤0.25 to >16	99.5	99.5
	Cefepime	≤0.25	>16	≤0.008 to >64	73.4	62.9
	Meropenem	0.03	0.12	≤0.004 to >64	94.4	95.0
North America (5835; 28.2%)	Cefepime–taniborbactam	0.06	0.12	≤0.25 to >16	99.9	99.9
	Cefepime	≤0.25	8	≤0.008 to 64	92.2	85.6
	Meropenem	0.03	0.12	≤0.004 to >64	98.3	98.6

^a^For comparative purposes only, % susceptible values for cefepime–taniborbactam correspond to the percentage of isolates inhibited at ≤16 mg/L.

^b^For cefepime tested against Enterobacterales isolates: percent susceptible values by CLSI breakpoints include isolates with both susceptible (MIC** **≤** **2 mg/L) and susceptible-dose dependent (MIC 4 and 8 mg/L) MICs.

### *P. aeruginosa*: phenotype analysis

Among the 7919 tested isolates of *P. aeruginosa*, 30.2/30.2% were imipenem resistant (by CLSI/EUCAST breakpoints), the cefepime–taniborbactam MIC_50_ and MIC_90_ were 2 and 8 mg/L, and 96.5% of isolates were inhibited by cefepime–taniborbactam at ≤16 mg/L (Table 6). The addition of taniborbactam to cefepime reduced the MIC_90_ value by 4-fold (from 32 mg/L to 8 mg/L). The most active comparators against *P. aeruginosa* overall were ceftazidime–avibactam (89.7% susceptible), ceftolozane–tazobactam (87.6% susceptible) and meropenem–vaborbactam (85.6% susceptible by EUCAST breakpoints).

**Table 6.** *In vitro* activity of cefepime–taniborbactam and comparator agents against 7919 clinical isolates of *P. aeruginosa*^a^

	MIC, mg/L			MIC interpretation					
				CLSI			EUCAST		
Antimicrobial agent	MIC_50_	MIC_90_	MIC range	% Susceptible	% Intermediate	% Resistant	% Susceptible	% Intermediate	% Resistant
Cefepime–taniborbactam^b^	2	8	≤0.06 to >32	96.5	NA^d^	3.5	96.5	NA	3.5
Cefepime^c^	4	32	≤0.25 to >32	78.4	8.8	12.7	78.4	NA	21.6
Ceftazidime–avibactam	2	16	≤0.25 to >16	89.7	NA	10.3	89.7	NA	10.3
Ceftazidime^c^	4	>32	≤0.25 to >32	75.8	4.9	19.3	75.8	NA	24.2
Ceftolozane–tazobactam	1	8	≤0.12 to >16	87.6	2.9	9.4	87.6	NA	12.4
Ciprofloxacin^c^	0.12	>4	≤0.06 to >4	74.9	4.1	21.0	74.9	NA	25.1
Gentamicin	2	>16	≤0.25 to >16	NA	NA	NA	NA	NA	NA
Imipenem^c^	2	16	≤0.5 to >64	58.4	11.3	30.2	69.8	NA	30.2
Meropenem–vaborbactam	0.5	16	≤0.06 to >16	NA	NA	NA	85.6	NA	14.4
Meropenem	0.5	>8	≤0.06 to >64	72.0	6.7	21.3	72.0	13.0	15.0
Piperacillin–tazobactam^c^	8	>128	≤0.5 to >128	70.6	7.8	21.6	70.6	NA	29.4

^a^The 7919 clinical isolates of *P. aeruginosa* were cultured from patients with community- and hospital-associated infections in 267 sites in 57 countries across five geographic regions (Asia/South Pacific, Europe, Latin America, Middle East/Africa and North America) in the years 2018 (*n *= 1343), 2019 (*n *= 1657), 2020 (*n *= 1617), 2021 (*n *= 1800) and 2022 (*n *= 1502). Patient locations/wards at the time of specimen collection included (*n*/percent of total): general medicine (2759/34.8%); medical ICU (1887/23.8%); general surgery (742/9.4%); ICU surgery (806/10.2%); emergency room (425/5.4%); paediatric ICU (373/4.7%); general ICU (436/5.5%); general paediatric (295/3.7%); and other/no location given (196/2.5%).

^b^For comparative purposes only, % susceptible and % resistant values for cefepime–taniborbactam correspond to the percentage of isolates inhibited at ≤16 mg/L and ≥32 mg/L, respectively.

^c^For cefepime, ceftazidime, ciprofloxacin, imipenem and piperacillin–tazobactam, percent susceptible values by EUCAST breakpoints include isolates in the susceptible, increased exposure category.

^d^NA, not available. Neither CLSI nor EUCAST publish MIC breakpoints for gentamicin against *P. aeruginosa*; CLSI does not publish MIC breakpoints for meropenem–vaborbactam tested against *P. aeruginosa*.

At ≤16 mg/L, cefepime–taniborbactam inhibited ≥80% of *P. aeruginosa* isolates that were resistant to meropenem, piperacillin–tazobactam and meropenem–vaborbactam; >70% of isolates resistant to cefepime, ceftazidime–avibactam, and ceftolozane–tazobactam; 80.2% of MDR isolates; and 73.0% of DTR isolates (Table 7). Cefepime–taniborbactam at ≤16 mg/L inhibited a greater percentage of isolates than ceftazidime–avibactam, meropenem–vaborbactam and ceftolozane–tazobactam for each of the nine resistance phenotypes analysed. Less than 60% of meropenem-resistant isolates, <50% of MDR isolates and <30% of DTR isolates were susceptible to ceftazidime–avibactam, meropenem–vaborbactam and ceftolozane–tazobactam.

**Table 7.** *In vitro* activity of cefepime–taniborbactam and comparator agents against clinical isolates of *P. aeruginosa* with antimicrobial-resistant phenotypes

		MIC, mg/L			MIC interpretation	
Antimicrobial-resistant phenotype, by prevalence (no. of isolates; % of total isolates)^a^	Antimicrobial agent	MIC_50_	MIC_90_	MIC range	CLSI % susceptible	EUCAST % susceptible
Piperacillin–tazobactam-resistant (1711; 21.6%)	Cefepime–taniborbactam^b^	8	64	0.5 to >128	86.1	86.1
	Ceftazidime–avibactam	8	>16	0.5 to >16	60.1	60.1
	Ceftolozane–tazobactam	4	>16	0.5 to >16	53.4	53.4
	Meropenem	8	64	≤0.06 to >64	32.1	32.1
	Meropenem–vaborbactam	8	>16	≤0.06 to >16	NA^c^	54.9
	Piperacillin–tazobactam^d^	>128	>128	64 to >128	0	0
Meropenem-resistant (1690; 21.3%)	Cefepime–taniborbactam	8	128	0.5 to >128	85.3	85.3
	Ceftazidime–avibactam	8	>16	0.5 to >16	58.5	58.5
	Ceftolozane–tazobactam	4	>16	0.5 to >16	53.4	53.4
	Meropenem	>8	>64	≤0.5 to >64	0	0
	Meropenem–vaborbactam	16	>16	0.5 to >16	NA	32.7
	Piperacillin–tazobactam	64	>128	1 to >128	21.0	21.0
MDR phenotype (1352; 17.1%)^e^	Cefepime–taniborbactam	8	128	0.5 to >128	80.2	80.2
	Ceftazidime–avibactam	16	>16	0.5 to >16	44.5	44.5
	Ceftolozane–tazobactam	16	>16	0.5 to >16	35.3	35.3
	Meropenem	>8	>64	0.12 to >64	8.7	8.7
	Meropenem–vaborbactam	16	>16	≤0.06 to >16	NA	36.7
	Piperacillin–tazobactam	128	>128	2 to >128	3.3	3.3
Meropenem–vaborbactam-resistant (1138; 14.4%)^f^	Cefepime–taniborbactam	8	>128	1 to >128	79.6	79.6
	Ceftazidime–avibactam	16	>16	1 to >16	44.8	44.8
	Ceftolozane–tazobactam	16	>16	0.5 to >16	41.9	41.9
	Meropenem	>8	>64	8 to >64	0	0
	Meropenem–vaborbactam	>16	>16	16 to >16	NA	0
	Piperacillin–tazobactam	64	>128	4 to >128	11.5	11.5
Cefepime-resistant (1008; 12.7%)	Cefepime–taniborbactam	8	>128	1 to >128	72.8	72.8
	Ceftazidime–avibactam	16	>16	0.5 to >16	34.0	34.0
	Ceftolozane–tazobactam	>16	>16	1 to >16	23.4	23.4
	Meropenem	>8	>64	0.12 to >64	18.4	18.4
	Meropenem–vaborbactam	16	>16	≤0.06 to >16	NA	39.0
	Piperacillin–tazobactam	>128	>128	2 to >128	3.6	3.6
DTR phenotype (825; 10.4%)^g^	Cefepime–taniborbactam	8	>128	1 to >128	73.0	73.0
	Ceftazidime–avibactam	>16	>16	0.5 to >16	28.1	28.1
	Ceftolozane–tazobactam	>16	>16	1 to >16	21.6	21.6
	Meropenem	>8	>64	4 to >64	0	0
	Meropenem–vaborbactam	>16	>16	0.5 to >16	NA	20.7
	Piperacillin–tazobactam	128	>128	32 to >128	0	0
Ceftazidime–avibactam-resistant (815; 10.3%)	Cefepime–taniborbactam	8	>128	1 to >128	71.3	71.3
	Ceftazidime–avibactam	>16	>16	16 to >16	0	0
	Ceftolozane–tazobactam	>16	>16	1 to >16	12.3	12.3
	Meropenem	>8	>64	0.25 to >64	8.5	8.5
	Meropenem–vaborbactam	>16	>16	0.25 to >16	NA	22.9
	Piperacillin–tazobactam	>128	>128	2 to >128	4.7	4.7
Ceftolozane–tazobactam-resistant (745; 9.4%)	Cefepime–taniborbactam	8	>128	0.5 to >128	70.1	70.1
	Ceftazidime–avibactam	>16	>16	1 to >16	18.4	18.4
	Ceftolozane–tazobactam	>16	>16	16 to >16	0	0
	Meropenem	>8	>64	≤0.06 to >64	6.3	6.3
	Meropenem–vaborbactam	>16	>16	≤0.06 to >16	NA	22.8
	Piperacillin–tazobactam	128	>128	2 to >128	6.3	6.3

^a^Resistant phenotypes determined using CLSI breakpoints except for meropenem–vaborbactam resistance (EUCAST; MIC** **≥** **16 mg/L). % of total isolates calculated as % of 7919 clinical isolates of *P. aeruginosa*.

^b^For comparative purposes only, % susceptible values for cefepime–taniborbactam correspond to the percentage of isolates inhibited at ≤16 mg/L.

^c^NA, not applicable (CLSI does not publish susceptible breakpoints for meropenem–vaborbactam against *P. aeruginosa*).

^d^For piperacillin–tazobactam, percent susceptible values by EUCAST breakpoints represent susceptible, increased exposure (MIC** **≤** **16 mg/L).

^e^An MDR phenotype was assigned to isolates resistant, using 2023 CLSI breakpoints,^8^ to at least one agent from ≥3 of the following antimicrobial agent classes: aminoglycosides (gentamicin), β-lactam combination agents (piperacillin–tazobactam, ceftazidime–avibactam, ceftolozane–tazobactam, meropenem–vaborbactam), carbapenems (meropenem or imipenem), cephems (ceftazidime, cefepime) and fluoroquinolones (levofloxacin or ciprofloxacin).

^f^Meropenem–vaborbactam-resistant phenotype based on EUCAST breakpoint (MIC** **≥** **16 mg/L).

^g^DTR isolates were identified using the definition of Kadri *et al*.^11^ as isolates intermediate or resistant, by 2023 CLSI breakpoints,^8^ to fluoroquinolones (levofloxacin) and all β-lactams including carbapenems and piperacillin–tazobactam, but excluding ceftazidime–avibactam, ceftolozane–tazobactam and meropenem–vaborbactam.

### *P. aeruginosa*: genotype analysis

In total, 1898 *P. aeruginosa* isolates were subjected to molecular testing by WGS or PCR and Sanger sequencing. Cefepime–taniborbactam was the most active agent against carbapenemase-producing subsets of *P. aeruginosa*, inhibiting 60.8% of all carbapenemase-positive isolates (MIC_50_, 8 mg/L; MIC_90_, >128 mg/L), 55.6% of all MBL-positive isolates (MIC_50_, 16 mg/L; MIC_90_, >128 mg/L) and 81.9% of VIM-positive isolates (MIC_50_, 8 mg/L; MIC_90_, 128 mg/L) (Table 8). Ceftazidime–avibactam inhibited only 12.7% of carbapenemase-positive *P. aeruginosa* isolates, and both ceftolozane–tazobactam and meropenem–vaborbactam were inactive. Table S7 depicts the cefepime–taniborbactam MIC distribution for the carbapenemase-positive *P. aeruginosa* isolates stratified by carbapenemase type. Among meropenem-resistant *P. aeruginosa*, 27.3% (460/1687) of isolates produced at least one of the queried carbapenemases. Against carbapenem-resistant *P. aeruginosa*, cefepime–taniborbactam inhibited 49%–60% more isolates with carbapenemases (*n *= 460) and 19%–51% more isolates without carbapenemases (*n *= 1227) than the other β-lactam/β-lactamase inhibitor combinations tested (Figure 1).

**Table 8.** *In vitro* activity of cefepime–taniborbactam and comparator agents against 1898 clinical isolates of *P. aeruginosa* with molecularly identified β-lactamase genotypes

		MIC, mg/L			MIC interpretation	
Genotype (no. of isolates; % of total molecularly characterized isolates)	Antimicrobial agent	MIC_50_	MIC_90_	MIC range	CLSI % susceptible	EUCAST % susceptible
Carbapenemase-positive (464; 24.4%)^a^	Cefepime–taniborbactam^b^	8	>128	0.5 to >128	60.8	60.8
	Ceftazidime–avibactam	>16	>16	2 to >16	12.7	12.7
	Ceftolozane–tazobactam	>16	>16	1 to >16	0.9	0.9
	Meropenem	>8	>64	2 to >64	0.2	0.2
	Meropenem–vaborbactam	>16	>16	1 to >16	NA^c^	5.6
	Piperacillin–tazobactam^d^	128	>128	2 to >128	4.1	4.1
MBL-positive (403; 21.2%)^e^	Cefepime–taniborbactam	16	>128	1 to >128	55.6	55.6
	Ceftazidime–avibactam	>16	>16	4 to >16	2.0	2.0
	Ceftolozane–tazobactam	>16	>16	1 to >16	0.7	0.7
	Meropenem	>8	>64	2 to >64	0.2	0.2
	Meropenem–vaborbactam	>16	>16	4 to >16	NA	5.5
	Piperacillin–tazobactam	128	>128	2 to >128	4.2	4.2
VIM-positive (271; 14.3%)^f^	Cefepime–taniborbactam	8	128	1 to >128	81.9	81.9
	Ceftazidime–avibactam	>16	>16	4 to >16	3.0	3.0
	Ceftolozane–tazobactam	>16	>16	1 to >16	1.1	1.1
	Meropenem	>8	>64	4 to >64	0	0
	Meropenem–vaborbactam	>16	>16	4 to >16	NA	5.9
	Piperacillin–tazobactam	64	>128	16 to >128	2.2	2.2
GES-positive (95; 5.0%)	Cefepime–taniborbactam	8	16	0.5 to >32	98.9	98.9
	Ceftazidime–avibactam	4	>16	2 to >16	64.2	64.2
	Ceftolozane–tazobactam	>16	>16	1 to >16	1.1	1.1
	Meropenem	>8	>64	0.5 to >64	2.1	2.1
	Meropenem–vaborbactam	>16	>16	0.25 to >16	NA	27.4
	Piperacillin–tazobactam	64	>128	8 to >128	5.3	5.3
VEB-positive (54; 2.8%)	Cefepime–taniborbactam	8	16	4 to 64	90.7	90.7
	Ceftazidime–avibactam	>16	>16	4 to >16	3.7	3.7
	Ceftolozane–tazobactam	>16	>16	>16 to >16	0	0
	Meropenem	>8	64	0.5 to >64	1.9	1.9
	Meropenem–vaborbactam	>16	>16	0.5 to >16	NA	3.7
	Piperacillin–tazobactam	128	>128	16 to >128	1.9	1.9
PER-positive (16; 0.8%)	Cefepime–taniborbactam	8	16	1 to 32	93.8	93.8
	Ceftazidime–avibactam	16	>16	4 to >16	12.5	12.5
	Ceftolozane–tazobactam	>16	>16	>16 to >16	0	0
	Meropenem	>8	32	4 to 32	0	0
	Meropenem–vaborbactam	16	>16	4 to >16	NA	25.0
	Piperacillin–tazobactam	32	>128	8 to >128	25.0	25.0
Carbapenemase-negative (1434; 75.6%)	Cefepime–taniborbactam	8	16	0.5 to >128	93.5	93.5
	Ceftazidime–avibactam	8	>16	≤0.25 to >16	76.6	76.6
	Ceftolozane–tazobactam	2	>16	0.5 to >16	73.9	73.9
	Meropenem	>8	16	≤0.06 to >64	10.9	10.9
	Meropenem–vaborbactam	8	>16	≤0.06 to >16	NA	51.3
	Piperacillin–tazobactam	64	>128	≤0.5 to >128	27.1	27.1

^a^Includes isolates harbouring GES-5, GES-6 and GES-20 (variants with reported carbapenemase activity).

^b^For comparative purposes only, % susceptible values for cefepime–taniborbactam correspond to the percentage of isolates inhibited at ≤16 mg/L.

^c^NA, not applicable (CLSI does not publish susceptible breakpoints for meropenem–vaborbactam against *P. aeruginosa*).

^d^For piperacillin–tazobactam, percent susceptible values by EUCAST breakpoints represent susceptible, increased exposure (MIC** **≤** **16 mg/L).

^e^Includes isolates harbouring VIM (*n *= 271), IMP (*n *= 62), NDM (*n *= 62), IMP + DIM (*n *= 2), IMP + VIM (*n *= 3), VIM + NDM (*n *= 1) and NDM + DIM (*n *= 2).

^f^Isolates could also possess serine carbapenemases, ESBLs, AmpCs and/or OSBLs, but no other MBLs.

### *P. aeruginosa*: characterization of isolates with elevated cefepime–taniborbactam MICs

Among the 560 *P. aeruginosa* isolates with elevated cefepime–taniborbactam MICs, 284 (51%) isolates had MICs of 16 mg/L and 276 (49%) had MICs** **>** **16 mg/L. Most *P. aeruginosa* isolates with cefepime–taniborbactam MICs** **>** **16 mg/L were MDR (268/276; 97.1%), and only 14.2%, 13.4% and 10.4% of these were susceptible to ceftazidime–avibactam, meropenem–vaborbactam (EUCAST breakpoint) and ceftolozane–tazobactam, respectively.

Among the 7919 isolates of *P. aeruginosa* tested, 560 (7.1%) had a cefepime–taniborbactam MIC of ≥16 mg/L and 557 of these were sequenced. Possible resistance mechanisms were identified for approximately 65% of sequenced isolates. Sixty-six (11.2%) of the 557 sequenced *P. aeruginosa* isolates with cefepime–taniborbactam MICs** **≥** **16 mg/L carried IMP (Table 9). One hundred and fifty-nine isolates (28.5%) had a PBP3 amino acid sequence that differed from the wild-type reference; of these, 85 isolates showed substitutions implicated in elevated resistance to cephalosporins.^16–18^ Alterations in, or absence of, genes coding for negative regulators of efflux systems (*esrC*, *mexR*, *mexS*, *mexT*, *mexZ*, *nalC*, *nalD*, *nfxB*)^19–22^ were identified in 53.5% (298/557) of isolates. Loss-of-function mutations in these loci are expected to result in up-regulation of multidrug efflux pumps in *P. aeruginosa*. The *oprD* gene exhibited a gross disruption expected to result in a non-functional porin (*n *= 379) or lacked the gene completely (*n *= 20) in 71.6% (399/557) of isolates. However, OprD deficiency is associated primarily with resistance to imipenem, not to cefepime and so may reflect prior carbapenem exposure in some of these isolates. Seventy-six of the 557 isolates (13.6%) exhibited a gross disruption in one or more of the regulatory genes (*ampD*, *dacB*, *mpl*, *ampR*), putatively resulting in overexpression of *Pseudomonas*-derived cephalosporinase (PDC), the intrinsic AmpC in *P. aeruginosa*.^16,18^ An additional 31 isolates were found to possess amino acid substitutions in AmpD that have been reported to lead to AmpC overexpression.^23–26^ Cefepime is only a weak inducer of AmpC and withstands hydrolysis by AmpC due to formation of a stable acyl-enzyme complex.^27^ Table 9 summarizes the overlap among putative resistance factors for *P. aeruginosa* isolates with elevated cefepime–taniborbactam MIC values, including IMP β-lactamases, *ftsI* mutations that code for PBP3 variants expected to result in elevated MICs for cefepime and other cephalosporins, and efflux-related genes with variations that potentially enhance efflux activity. Among isolates with cefepime–taniborbactam MIC values of 16 mg/L that were sequenced (*n *= 282), up-regulated efflux was the sole putative resistance mechanism observed in approximately 60% of isolates. No isolates in this group carried an IMP MBL. In contrast, IMP-producers, with or without PBP3 sequence variation and/or efflux up-regulation, accounted for 24.0% (66/275) of the sequenced isolates that tested with cefepime–taniborbactam MIC values** **>** **16 mg/L. Potential resistance mechanisms were not observed in 30.5% and 40.4% of *P. aeruginosa* isolates with cefepime–taniborbactam MICs of 16 mg/L and >16 mg/L, respectively.

**Table 9.** Occurrence and co-occurrence of putative resistance mechanisms identified in 557 isolates of *P. aeruginosa* with cefepime–taniborbactam MICs** **≥** **16 mg/L

	Cefepime–taniborbactam MIC (number of isolates)	
	16 mg/L (*n *= 282)	>16 mg/L (*n* = 275)
Putative resistance mechanism(s)^a^	Number of isolates (%)	Number of isolates (%)
Efflux up-regulation alone	167 (59.2)	53 (19.3)
Efflux up-regulation and PBP3 sequence variation	16 (5.7)	28 (10.2)
PBP3 sequence variation alone	13 (4.6)	18 (6.5)
IMP-producer alone	0	29 (10.5)
IMP-producer and efflux up-regulation	0	26 (9.5)
IMP-producer and PBP3 sequence variation and efflux up-regulation	0	8 (2.9)
IMP-producer and PBP3 sequence variation	0	3 (1.1)
No putative resistance mechanism(s) identified	86 (30.5)	111 (40.4)

^a^IMP is outside the spectrum of taniborbactam inhibition; PBP3 sequence variation included *ftsI* mutations that code for PBP3 variants with reduced cefepime binding, including G63D, G216S, A244T, R504C, I524T, P527S, G531D and F533L^16–18^; and efflux up-regulation included variation or absence of efflux-related regulatory genes including *esrC*, *mexR*, *mexS*, *mexT*, *mexZ*, *nalC*, *nalD* and *nfxB* that likely enhance drug extrusion.^19–22^

### *P. aeruginosa*: regional, specimen source and species analyses

**Figure 3.**
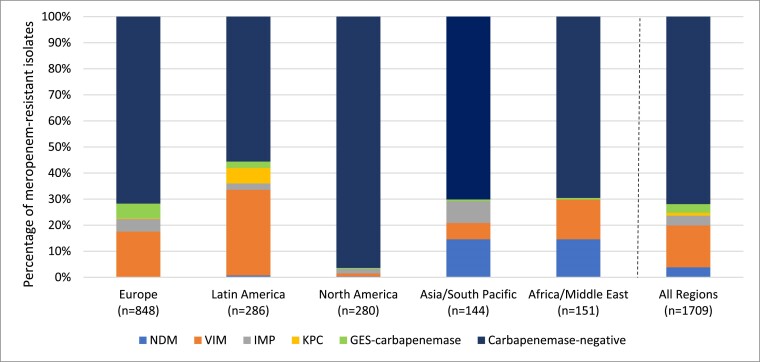
Global region diversity of carbapenemases detected among meropenem-resistant *P. aeruginosa*^a^. ^a^Isolates that carried multiple carbapenemases were counted for each carbapenemase type. This figure appears in colour in the online version of *JAC* and in black and white in the print version of *JAC*.

Meropenem susceptible values were <80% in each of the five global regions studied, ranging from 77.9%–78.9% in North America and Asia/South Pacific to 66.0%–68.3% in Latin America and Europe (Table 10). Cefepime–taniborbactam at ≤16 mg/L inhibited from 94.4% (Asia/South Pacific) to 97.4% (North America) of *P. aeruginosa* across the five global regions. For all regions, carbapenemase-negative isolates were encountered more frequently (>50%) than those carrying a carbapenemase (Figure 3). Among isolates with detected carbapenemases, VIM was most frequently observed in Europe, Latin America and Africa/Middle East. NDM was most frequently observed in isolates from the Asia/South Pacific region. In North America, IMP was most frequently detected; however, only 10 isolates in total carried carbapenemases. Across five common specimen sources, limited variation (3%) was observed in the percentage of isolates with cefepime–taniborbactam MICs of ≤16 mg/L (94.2%–97.2%) (Table S8).

**Table 10.** *In vitro* activity of cefepime–taniborbactam, cefepime, and meropenem against 7919 clinical isolates of *P. aeruginosa* stratified by global region of collection

		MIC, mg/L			MIC interpretation	
	Antimicrobial agent	MIC_50_	MIC_90_	MIC range	CLSI % susceptible	EUCAST % susceptible
Region of collection (no. of isolates; % of total isolates)						
Asia/South Pacific (896; 11.3%)	Cefepime–taniborbactam^a^	2	8	≤0.06 to >128	94.4	94.4
	Cefepime^b^	2	32	≤0.25 to >32	83.6	83.6
	Meropenem	0.5	>8	≤0.06 to >64	78.9	78.9
Europe (3480; 43.9%)	Cefepime–taniborbactam	2	8	≤0.06 to >128	97.2	97.2
	Cefepime	4	32	≤0.25 to >32	77.4	77.4
	Meropenem	0.5	>8	≤0.06 to >64	68.3	68.3
Latin America (983; 12.4%)	Cefepime–taniborbactam	2	8	0.12 to >128	95.1	95.1
	Cefepime	4	32	≤0.25 to >32	74.7	74.7
	Meropenem	0.5	>8	≤0.06 to >64	66.0	66.0
Middle East/Africa (774; 9.8%)	Cefepime–taniborbactam	2	8	0.25 to >128	95.6	95.6
	Cefepime	4	32	≤0.25 to >32	78.4	78.4
	Meropenem	0.5	>8	≤0.06 to >64	74.7	74.7
North America (1786; 22.6%)	Cefepime–taniborbactam	2	8	≤0.06 to >128	97.4	97.4
	Cefepime	4	32	≤0.25 to >32	80.1	80.1
	Meropenem	0.5	8	≤0.06 to >64	77.9	77.9

^a^For comparative purposes only, % susceptible values for cefepime–taniborbactam correspond to the percentage of isolates inhibited at ≤16 mg/L.

^b^For cefepime tested against *P. aeruginosa* isolates, percent susceptible values by EUCAST breakpoints correspond to isolates with susceptible, increased exposure (MIC** **≤** **8 mg/L) MICs.

## Discussion

Globally, carbapenem resistance in Enterobacterales is mediated primarily by carbapenemases (current study, 93%) although regional variation exists.^28^ Carbapenemase carriage among meropenem-resistant Enterobacterales in the present study ranged from 75% in North America to 96% in Asia/South Pacific region (Figure 2). Carbapenemase genes, both serine carbapenemases and MBLs, are present on plasmids and integrons, and have spread globally. Greater dissemination of carbapenemase genes is anticipated worldwide.

The epidemiology of carbapenemases is heterogeneous. Among meropenem-resistant Enterobacterales, KPC is the most common carbapenemase in Europe, Latin America and North America; NDM is most common in Asia/South Pacific and Africa/Middle East regions; and OXA-48-like is most common in Asia/South Pacific, Africa/Middle East and European regions (Figure 2). In the current study, cefepime–taniborbactam at ≤16 mg/L inhibited ≥98% of Enterobacterales in all five global regions studied coinciding with the rarity of IMP-positive isolates, and other potential cefepime–taniborbactam resistance mechanisms, in clinical isolates in each region. Cefepime–taniborbactam at ≤16 mg/L inhibited ≥94% of *P. aeruginosa* in all five global regions (Table 10). Carbapenem resistance is more common in *P. aeruginosa* (current study 15%–21%) than Enterobacterales (current study 4%–5%). Globally, 27.3% of carbapenem-resistant *P. aeruginosa* isolates carried a carbapenemase (range, 4% in North America to 45% in Latin America) (Figure 3), confirming data from other sources.^29,30^ Overall, the current study found that VIM was the most common carbapenemase identified in carbapenem-resistant *P. aeruginosa* isolates (59% of carbapenemase-positive isolates carried VIM either alone or in combination with other carbapenemases).

Taniborbactam restored susceptibility to cefepime for the majority of global clinical isolates of cephalosporin-resistant, carbapenem-resistant, MDR, DTR, ceftolozane–tazobactam-resistant, ceftazidime–avibactam-resistant and meropenem–vaborbactam-resistant Enterobacterales and *P. aeruginosa*, confirming results published previously in isolate limited and regional studies.^2,3,10,31–38^ The *in vitro* activity of cefepime–taniborbactam included isolates of Enterobacterales and *P. aeruginosa* carrying NDM and VIM MBLs and common serine carbapenemases (KPC, OXA-48-like, GES) as well as carbapenemase-negative isolates with carbapenem-resistant phenotypes.

Cefepime–taniborbactam MICs of ≥16 mg/L were identified in 0.7% (153/20 725) of Enterobacterales and 7.1% (560/7919) of *P. aeruginosa* isolates tested. We identified multiple and often concurrent potential mechanisms of resistance, including carriage of IMP, alterations in PBP3 (*ftsI*), porins (decreased permeability) and efflux (up-regulation) in these isolates confirming earlier reports.^2,3,31,35–39^ However, possible resistance mechanisms were only identified in approximately 65% of *P. aeruginosa* tested suggesting additional efforts are needed to definitively link phenotype to genotype amongst *P. aeruginosa* with cefepime–taniborbactam MICs** **≥** **16 mg/L. The further testing could involve RNA expression profiling of resistance genes; western blotting for porin and efflux pump protein expression; and WGS. These types of analyses would also need to be performed on isolates in the provisional susceptible range for cefepime–taniborbactam to avoid incorrect conclusions about phenotypic–genotypic relationships.

Rare NDM (NDM-9, NDM-30) and VIM (VIM-83) variants in *E. coli* or *K. pneumoniae* with single amino acid substitutions that disrupt the electrostatic interaction of negative charges in the active site loops of MBLs with the *N*-(2-aminoethyl)cyclohexylamine side chain of taniborbactam have been reported.^1,40–42^ NDM-30 or VIM-83 was not identified in any clinical isolates of Enterobacterales or *P. aeruginosa* in the current study, and only one isolate carrying NDM-9 (*K. pneumoniae* from Philippines; cefepime–taniborbactam MIC = 64 mg/L) was found among the 28 644 isolates from 336 hospitals in 59 countries from 2018 to 2022, supporting the present rarity of these specific MBLs. While the potential emergence of NDM and VIM variants that confer resistance to cefepime–taniborbactam will need to be closely monitored as it is introduced into clinical use, cefepime–taniborbactam demonstrates potent *in vitro* activity against isogenic strains of *E. coli* overproducing NDM variants NDM-1, NDM-2, NDM-4, NDM-5, NDM-7, NDM-14, NDM-19, NDM-30, NDM-35 and NDM-47; VIM variants VIM-1, VIM-2, VIM-4, VIM-5, VIM-6, VIM-19 and VIM-53; as well as SIM-1, SPM-1, GIM-1, DIM-1, PFM-1 and AIM-1 MBLs.^2,41^

The current study summarizes *in vitro* data for the largest collection of clinical Gram-negative isolates tested against cefepime–taniborbactam to date (>28 000 isolates). Once it is approved for clinical use, cefepime–taniborbactam could augment the few existing agents or combinations with activity against MDR Enterobacterales and *P. aeruginosa* including most isolates producing NDM and VIM MBLs. Currently, there are no approved β-lactam/β-lactamase inhibitor combinations indicated for the treatment of infections caused by MBL-producing Gram-negative bacilli. Cefepime–taniborbactam is also highly active against isolates carrying serine carbapenemases and carbapenemase-resistant isolates that are carbapenemase-negative. This report may serve as a baseline to monitor future changes in the susceptibility of Gram-negative species to cefepime–taniborbactam following its introduction into clinical use. Continued surveillance of cefepime–taniborbactam is warranted.

## Supplementary Material

dkae329_Supplementary_Data

## Supplementary data

Tables S1–S8 are available as Supplementary data at *JAC* Online.
