# Supplementary material for: Cefepime–taniborbactam activity against antimicrobial-resistant clinical isolates of Enterobacterales and Pseudomonas aeruginosa: GEARS global surveillance programme 2018–22
Source: J Antimicrob Chemother. 2024 Sep 17;79(12):3116–31. doi: 10.1093/jac/dkae329 (PMC11638001; doi:10.1093/jac/dkae329)
Supplement: dkae329_Supplementary_Data [file dkae329_supplementary_data.zip › JAC-2024-0731_R1_Supplementary_Data_Yellow_Clean.docx]

**Supplementary data**

**Table S1.** Total isolate counts by group/species for the 20725 Enterobacterales isolates collected by the GEARS Global Surveillance Program from 2018 to 2022

| Group/Species | Number of isolates | Percentage of total Enterobacterales isolates |
| --- | --- | --- |
| *Citrobacter freundii* complex: | 1062 | 5.1% |
| *Citrobacter braakii* | 54 | 0.3% |
| *Citrobacter freundii* | 1001 | 4.8% |
| *Citrobacter sedlakii* | 6 | <0.1% |
| *Citrobacter youngae* | 1 | <0.1% |
| *Citrobacter amalonaticus* | 9 | <0.1% |
| *Citrobacter farmeri* | 4 | <0.1% |
| *Citrobacter koseri* | 590 | 2.8% |
| *Citrobacter* sp. (unable to speciate) | 9 | <0.1% |
| *Enterobacter cloacae* complex: | 1314 | 6.3% |
| *Enterobacter asburiae* | 55 | 0.3% |
| *Enterobacter bugandensis* | 80 | 0.4% |
| *Enterobacter cloacae* | 904 | 4.4% |
| *Enterobacter cloacae* complex^a^ | 58 | 0.3% |
| *Enterobacter hormaechei* | 163 | 0.8% |
| *Enterobacter kobei* | 27 | 0.1% |
| *Enterobacter ludwigii* | 27 | 0.1% |
| *Enterobacter roggenkampii* | 13 | <0.1% |
| *Enterobacter xiangfangensis* | 24 | 0.1% |
| *Enterobacter* sp. (unable to speciate) | 179 | 0.9% |
| *Escherichia coli* | 5717 | 28.2% |
| *Klebsiella aerogenes* | 606 | 2.9% |
| *Klebsiella oxytoca* | 1094 | 5.3% |
| *Klebsiella pneumoniae* | 5700 | 27.5% |
| *Klebsiella variicola* | 4 | <0.1% |
| *Morganella morganii* | 634 | 3.1% |
| *Proteus mirabilis* | 1435 | 6.9% |
| *Proteus vulgaris* | 588 | 2.8% |
| *Proteus* sp. (unable to speciate) | 5 | <0.1% |
| *Providencia alcalifaciens* | 4 | <0.1% |
| *Providencia rettgeri* | 253 | 1.2% |
| *Providencia rustigianii* | 1 | <0.1% |
| *Providencia stuartii* | 335 | 1.6% |
| *Providencia* sp. (unable to speciate) | 12 | <0.1% |
| *Serratia fonticola* | 1 | <0.1% |
| *Serratia liquefaciens* | 64 | 0.3% |
| *Serratia marcescens* | 757 | 3.7% |
| *Serratia odorifera* | 2 | <0.1% |
| *Serratia rubidaea* | 5 | <0.1% |
| *Serratia ureilytica* | 24 | 0.1% |
| *Serratia* sp. (unable to speciate) | 280 | 1.4% |

^a^ Isolates for which MALDI-TOF analysis identified only to complex level.

**Table S2.** WGS and β-lactamase characterization via PCR and Sanger sequencing methodologies

| MDR and DTR definitions |
| --- |
| An MDR phenotype was assigned to isolates resistant, using 2023 CLSI breakpoints,^1^ to at least one agent from ≥3 of the following antimicrobial agent classes: aminoglycosides (gentamicin), β-lactam combination agents (piperacillin-tazobactam, ceftazidime-avibactam, ceftolozane-tazobactam, or meropenem-vaborbactam), carbapenems (meropenem or imipenem), cephems (ceftazidime or cefepime), and fluoroquinolones (levofloxacin or ciprofloxacin). Difficult-to-treat-resistant (DTR) isolates were identified using the definition of Kadri *et al*.^2^ as isolates intermediate or resistant, by 2023 CLSI breakpoints,^1^ to fluoroquinolones (levofloxacin) and all β-lactams including carbapenems and piperacillin-tazobactam, but excluding ceftazidime-avibactam, ceftolozane-tazobactam, and meropenem-vaborbactam. |
| WGS methodology |
| Cells were pelleted from 3 mL liquid cultures grown overnight from one colony in Brain Heart Infusion broth (Sigma-Aldrich, St. Louis, MO, USA) at 37°C with shaking. DNA was subsequently extracted using the DNeasy Ultraclean Microbial extraction kit (Qiagen, Germantown, MD, USA). Sequencing libraries were prepared using a NEBNext Ultra II (New England Biolabs, Ipswich, MA, USA) library preparation kit. Sequencing was performed on an Illumina Hiseq system using 2×150 bp paired end reads with a target coverage depth of 100×.  All analyses were carried out using the CLC Genomics Workbench, version 20 (Qiagen). For resistance gene identification, *de novo* assemblies of each genome were queried using the find resistance module, which interrogates the CGE database for resistance genes.^3^ To better detect highly diverse *ampC* genes for which there are few variants defined by CGE, the thresholds for minimum nucleotide sequence identity and minimum sequence length were set to 72% and 80%, respectively. β-lactamase genes with <100% sequence identity to a known nucleotide reference were translated to their deduced amino acid sequence and BLAST searched against the Refseq database in Genbank dedicated to β-lactamase nomenclature (Bioproject 313047) in order to assign the variant of the gene.  For porin gene identification, specified porin genes (Table S3) were searched by tBLASTn in the *de novo* assemblies of the genomes and screened for lesions. Gross disruptions were defined as any mutation that caused a stop codon to be read in-frame upstream of the stop codon in the reference sequence. Additionally, if two parts of a porin gene were on different contigs and interspaced by an insertion sequence (as identified by BLAST search in the Genbank nr/nt database), the porin gene was considered to be disrupted.  For *ftsI* (encoding penicillin-binding protein 3, PBP3), efflux pump regulatory gene and other gene-specific analysis, protein reference sequences (Table S3) were BLAST searched on a species-specific basis in *de novo* assemblies of each genome. In brief, tBLASTn was used to find the gene with the lowest E value in each assembly (database) to the reference sequence (query), for which mutations encoding amino acid changes were identified and reported. |
| β-lactamase characterization via PCR and Sanger sequencing methodology |
| For PCR/Sanger, Enterobacterales isolates and *P. aeruginosa* were screened for the presence of acquired *bla* encoding ESBLs (TEM, SHV, CTX-M, GES, VEB and PER), serine carbapenemases (KPC, OXA-48 group [Enterobacterales only)], OXA-24 group [*P. aeruginosa* only), metallo-β-lactamases (NDM, IMP, VIM, SPM, GIM), and AmpC β-lactamases (ACC, ACT, CMY, DHA, FOX, MIR, MOX) by multiplex PCR using published primers.^4^ Detected genes were amplified with extragenic primers and sequenced in full except for *bla*_TEM_ and *bla*_SHV_. These genes were screened by limited sequencing to identify those encoding TEM-type and SHV-type enzymes containing amino acid substitutions common to ESBLs at the following positions (Ambler numbering): SHV amino acids 146, 179, 238, 240; TEM amino acids 104, 164, 238, 240. For all fully sequenced β-lactamase genes, the deduced amino acid sequence was compared to available databases maintained by the NCBI (www.ncbi.nlm.nih.gov) to identify curated enzyme variants. |

**References**

1. CLSI. Performance Standards for Antimicrobial Susceptibility Testing-Thirty-Third Edition: M100. 2023.
2. Kadri SS, Adjemian J, Lai YL *et al*. Difficult-to-treat resistance in Gram-negative bacteremia at 173 US hospitals: retrospective cohort analysis of prevalence, predictors, and outcome of resistance to all first-line agents. *Clin Infect Dis* 2018; **67:** 1803-14. <https://doi.10.1093/cid/ciy378>
3. Bortolaia V, Kass RS, Ruppe E *et al*. ResFinder 4.0 for predictions of phenotypes and genotypes. *J Antimicrob Chemother* 2020; **75:** 3491-500. https://doi:10.1093/jac/dkaa345
4. Lob SH, Biedenbach DJ, Badal RE *et al*. Antimicrobial resistance and resistance mechanisms of *Enterobacteriaceae* in ICU and non-ICU wards in Europe and North America: SMART 2011-2013. *J Glob Antimicrob Resist* 2015; **3:** 190-7. <https://doi>:10.1016/j.jgar.2015.05.005

**Table S3.** Reference genes used in WGS analysis

| Organism | Strain | Gene name | NT accession | Locus tag/CDS | AA accession |
| --- | --- | --- | --- | --- | --- |
| *Citrobacter freundii* | ATCC 8090 | *ompC* | NZ_JMTA01000004 | GCFR_RS19300 | WP_003839420.1 |
| *Citrobacter freundii* | ATCC 8090 | *ompF* | NZ_JMTA01000013 | GCFR_RS14345 | WP_016149871.1 |
| *Citrobacter freundii* | ATCC 8090 | *ftsI* | NZ_JMTA01000025 | GCFR_RS10160 | WP_003018783.1 |
| *Enterobacter cloacae* | ATCC 13047 | *ompC* | NC_014121 | ECL_03519 | YP_003614002.1 |
| *Enterobacter cloacae* | ATCC 13047 | *ompF* | NC_014121 | ECL_02724 | YP_003613214.1 |
| *Enterobacter cloacae* | ATCC 13047 | *ftsI* | NC_014121 | ECL_00881 | YP_003611393.1 |
| *Escherichia coli* | K-12 MG1655 | *ompC* | NC_000913.3 | b2215 | NP_416719.1 |
| *Escherichia coli* | K-12 MG1655 | *ompF* | NC_000913.3 | b0929 | NP_415449.1 |
| *Escherichia coli* | K-12 MG1655 | *ftsI* | NC_000913.3 | b0084 | AIL15882.1 |
| *Escherichia coli* | K-12 MG1655 | *marR* | NC_000913.3 | b1530 | NP_416047 |
| *Escherichia coli* | K-12 MG1655 | *marA* | NC_000913.3 | b1531 | NP_416048 |
| *Escherichia coli* | K-12 MG1655 | *marB* | NC_000913.3 | b1532 | NP_416049 |
| *Escherichia coli* | K-12 MG1655 | *acrR* | NC_000913.3 | b0464 | NP_414997 |
| *Escherichia coli* | K-12 MG1655 | *acrA* | NC_000913.3 | b0463 | NP_414996 |
| *Escherichia coli* | K-12 MG1655 | *acrB* | NC_000913.3 | b0462 | NP_414995 |
| *Escherichia coli* | K-12 MG1655 | *ompR* | NC_000913.3 | b3405 | NP_417864 |
| *Escherichia coli* | K-12 MG1655 | *envZ* | NC_000913.3 | b3404 | NP_417863 |
| *Klebsiella pneumoniae* | ATCC 13883 | *ompK35* | NZ_KN046818 | DR88_RS17700 | WP_004141771.1 |
| *Klebsiella pneumoniae* | ATCC 13883 | *ompK36* | NZ_KN046818 | DR88_RS05545 | WP_004149145.1 |
| *Klebsiella pneumoniae* | ATCC 13883 | *ftsI* | NZ_KN046818 | DR88_RS22130 | WP_002888559.1 |
| *Klebsiella pneumoniae* | ATCC 13883 | *ramR* | NZ_KN046818 | DR88_RS19865 | WP_002893026.1 |
| *Pseudomonas aeruginosa* | PAO1 | *oprD* | NC_002516.2 | PA0958 | NP_249649.1 |
| *Pseudomonas aeruginosa* | PAO1 | *ftsI* | NC_002516.2 | PA4418 | NP_253108.1 |
| *Pseudomonas aeruginosa* | PAO1 | *mexR* | NC_002516.2 | PA0424 | NP_249115.1 |
| *Pseudomonas aeruginosa* | PAO1 | *mexS* | NC_002516.2 | PA2491 | NP_251181.1 |
| *Pseudomonas aeruginosa* | PAO1 | *mexT* | AJ007825 | N/A | CAA07694.1 |
| *Pseudomonas aeruginosa* | PAO1 | *mexZ* | NC_002516.2 | PA2020 | NP_250710.1 |
| *Pseudomonas aeruginosa* | PAO1 | *nalC* | NC_002516.2 | PA3721 | NP_252410.1 |
| *Pseudomonas aeruginosa* | PAO1 | *nalD* | NC_002516.2 | PA3574 | NP_252264.1 |
| *Pseudomonas aeruginosa* | PAO1 | *nfxB* | NC_002516.2 | PA4600 | NP_253290.1 |
| *Pseudomonas aeruginosa* | PAO1 | *esrC* | NC_002516.2 | PA4596 | NP_253286.1 |
| *Pseudomonas aeruginosa* | PAO1 | *mpl* | NC_002516.2 | PA4020 | NP_252709.1 |
| *Pseudomonas aeruginosa* | PAO1 | *dacB* | NC_002516.2 | PA3047 | NP_251737.1 |
| *Pseudomonas aeruginosa* | PAO1 | *ampR* | NC_002516.2 | PA4109 | NP_252798.1 |
| *Pseudomonas aeruginosa* | PAO1 | *ampD* | NC_002516.2 | PA4522 | NP_253212.1 |
| *Providencia rettgeri* | Pr-15-2-5 | *ompF-like* | NZ_CP039844 | FCT01_RS14495 | WP_137019368.1 |
| *Providencia rettgeri* | Pr-15-2-5 | *ftsI* | NZ_CP039844 | FCT01_RS06535 | WP_137018924.1 |
| *Serratia marcescens* | UMH2 | *ompC-like* | NZ_CP018924 | BVG88_RS14160 | WP_033643836.1 |
| *Serratia marcescens* | UMH2 | *oprD* | NZ_CP018924 | BVG88_RS02820 | WP_033642176.1 |
| *Serratia marcescens* | UMH2 | *ftsI* | NZ_CP018924 | BVG88_RS00340 | WP_004932790.1 |

**Table S4.** Cefepime-taniborbactam MIC distribution for 961 isolates of carbapenemase-positive Enterobacterales^a^ stratified by carbapenemase type

|  | Cefepime-taniborbactam MIC, mg/L; number of isolates (cumulative % of isolates inhibited at MIC) | | | | | | | | | | |  |
| --- | --- | --- | --- | --- | --- | --- | --- | --- | --- | --- | --- | --- |
| Carbapenemase | ≤0.03 | 0.06 | 0.12 | 0.25 | 0.5 | 1 | 2 | 4 | 8 | 16 | ≥32 | Total |
| KPC^b^ | 16 (4.9) | 37 (16.2) | 44 (29.7) | 48 (44.3) | 36 (55.4) | 49 (70.3) | 54 (86.9) | 30 (96.0) | 13 (100) |  |  | 327 |
| NDM^c^ |  | 1 (0.3) | 7 (2.2) | 19 (7.4) | 63 (24.7) | 69 (43.7) | 43 (55.5) | 13 (59.1) | 26 (66.2) | 34 (75.5) | 89 (100) | 364 |
| OXA-48-like^d^ | 2 (0.9) | 2 (1.8) | 16 (8.8) | 22 (18.4) | 18 (26.3) | 38 (43.0) | 55 (67.1) | 57 (92.1) | 14 (98.2) | 1 (98.7) | 3 (100) | 228 |
| VIM^c^ | 1 (2.4) | 0 (2.4) | 3 (9.5) | 4 (19.0) | 7 (35.7) | 12 (64.3) | 4 (73.8) | 5 (85.7) | 2 (90.5) | 2 (95.2) | 2 (100) | 42 |
| Total (cumulative % inhibited^e^) | 19 (2.0) | 40 (6.1) | 70 (13.4) | 93 (23.1) | 124 (36.0) | 168 (53.5) | 156 (69.7) | 105 (80.6) | 55 (86.4) | 37 (90.2) | 94 (100) | 961 |

^a^ IMP-positive isolates (*n*=7) were excluded from the dataset as IMP is outside the spectrum of taniborbactam inhibition.

^b^ Isolates could also possess OXA-48 group, ESBLs, AmpC-type enzymes and/or OSBLs (original-spectrum β-lactamases), but not MBLs.

^c^ Isolates could also possess serine carbapenemases, ESBLs, AmpC-type enzymes and/or OSBLs, but no other MBLs.

^d^ Isolates could also possess ESBLs, AmpC-type enzymes and/or OSBLs, but no other carbapenemases.

^e^ Cumulative % of all carbapenemase-positive isolates inhibited at cefepime-taniborbactam MIC.

**Table S5.** *In vitro* activity of cefepime-taniborbactam, cefepime, and meropenem against 20725 clinical isolates of Enterobacterales stratified by specimen source

|  |  | MIC, mg/L | | |  | MIC interpretation | | |
| --- | --- | --- | --- | --- | --- | --- | --- | --- |
|  | Antimicrobial agent | MIC_50_ | MIC_90_ | MIC range |  | CLSI % susceptible |  | EUCAST % susceptible |
| Specimen source  (no. of isolates; % of total isolates)^a^ |  |  |  |  |  |  |  |  |
| Blood (2913; 14.1%) | Cefepime-taniborbactam^b^ | 0.06 | 0.25 | ≤0.008 – 32 |  | 99.8 |  | 99.8 |
|  | Cefepime^c^ | ≤0.25 | >16 | ≤0.25 – >16 |  | 80.7 |  | 72.1 |
|  | Meropenem | 0.03 | 0.12 | ≤0.004 – >64 |  | 93.5 |  | 94.3 |
| Intraabdominal (3474; 16.8%) | Cefepime-taniborbactam | 0.06 | 0.25 | ≤0.008 – 32 |  | 99.6 |  | 99.6 |
|  | Cefepime | ≤0.25 | >16 | ≤0.25 – >16 |  | 86.6 |  | 79.0 |
|  | Meropenem | 0.03 | 0.12 | ≤0.004 – >64 |  | 95.3 |  | 96.0 |
| Respiratory (7408; 35.7%) | Cefepime-taniborbactam | 0.06 | 0.25 | ≤0.008 – >64 |  | 99.3 |  | 99.3 |
|  | Cefepime | ≤0.25 | >16 | ≤0.25 – >16 |  | 82.9 |  | 75.5 |
|  | Meropenem | 0.03 | 0.12 | ≤0.004 – >64 |  | 93.7 |  | 94.2 |
| Skin and soft tissue (1206; 5.8%) | Cefepime-taniborbactam | 0.06 | 0.12 | ≤0.008 – >16 |  | 99.4 |  | 99.4 |
|  | Cefepime | ≤0.25 | >16 | ≤0.25 – >16 |  | 86.4 |  | 81.1 |
|  | Meropenem | 0.06 | 0.12 | 0.008 – >64 |  | 95.5 |  | 95.9 |
| Urine (5722; 27.6%) | Cefepime-taniborbactam | 0.06 | 0.25 | ≤0.008 – >64 |  | 99.5 |  | 99.5 |
|  | Cefepime | ≤0.25 | >16 | ≤0.25 – >16 |  | 85.5 |  | 78.5 |
|  | Meropenem | 0.03 | 0.12 | ≤0.004 – >64 |  | 96.2 |  | 96.7 |

^a^ Two Enterobacterales isolates did not have a specimen source assigned.

^b^ For comparative purposes only, % susceptible values for cefepime-taniborbactam correspond to the percentage of isolates inhibited at ≤16 mg/L.

^c^ For cefepime tested against Enterobacterales isolates: percent susceptible values by CLSI breakpoints include isolates with both susceptible (MIC ≤2 mg/L) and susceptible-dose dependent (MIC 4 and 8 mg/L) MICs.

**Table S6.** *In vitro* activity of cefepime-taniborbactam, cefepime, and meropenem against individual species of Enterobacterales

|  |  | MIC, mg/L | | |  | MIC interpretation | | |
| --- | --- | --- | --- | --- | --- | --- | --- | --- |
| Species^a^ (no. of isolates) | Antimicrobial agent | MIC_50_ | MIC_90_ | MIC range |  | CLSI % susceptible |  | EUCAST % susceptible |
| *Citrobacter* *braakii* (54) | Cefepime-taniborbactam^b^ | 0.03 | 0.12 | 0.015 – 1 |  | 100 |  | 100 |
|  | Cefepime^c^ | ≤0.25 | 1 | ≤0.25 – 8 |  | 100 |  | 90.7 |
|  | Meropenem | 0.03 | 0.06 | ≤0.004 – 1 |  | 100 |  | 100 |
| *Citrobacter* *freundii* (1001) | Cefepime-taniborbactam | 0.03 | 0.12 | ≤0.008 – 32 |  | 99.8 |  | 99.8 |
|  | Cefepime | ≤0.25 | 2 | ≤0.25 – >16 |  | 92.6 |  | 84.8 |
|  | Meropenem | 0.03 | 0.12 | ≤0.004 – >64 |  | 97.2 |  | 97.9 |
| *Citrobacter* *koseri* (590) | Cefepime-taniborbactam | 0.03 | 0.12 | ≤0.008 – 2 |  | 100 |  | 100 |
|  | Cefepime | ≤0.25 | ≤0.25 | ≤0.25 – >16 |  | 98.8 |  | 97.8 |
|  | Meropenem | 0.03 | 0.06 | ≤0.004 – 8 |  | 99.5 |  | 99.7 |
| *Enterobacter* *asburiae* (55) | Cefepime-taniborbactam | 0.03 | 0.12 | ≤0.008 – 0.25 |  | 100 |  | 100 |
|  | Cefepime | ≤0.25 | 0.5 | ≤0.25 – 16 |  | 98.2 |  | 96.4 |
|  | Meropenem | 0.06 | 0.12 | 0.015 – 1 |  | 100 |  | 100 |
| *Enterobacter* *bugandensis* (80) | Cefepime-taniborbactam | 0.06 | 0.06 | 0.015 – 0.5 |  | 100 |  | 100 |
|  | Cefepime | ≤0.25 | ≤0.25 | ≤0.25 – 2 |  | 100 |  | 98.8 |
|  | Meropenem | 0.06 | 0.12 | 0.015 – 0.5 |  | 100 |  | 100 |
| *Enterobacter* *cloacae* (904) | Cefepime-taniborbactam | 0.06 | 0.5 | ≤0.008 – 32 |  | 99.8 |  | 99.8 |
|  | Cefepime | ≤0.25 | 16 | ≤0.25 – >16 |  | 88.4 |  | 70.7 |
|  | Meropenem | 0.03 | 0.25 | ≤0.004 – >64 |  | 95.1 |  | 95.9 |
| *Enterobacter* *cloacae* complex^d^ (58) | Cefepime-taniborbactam | 0.06 | 0.5 | 0.015 – 2 |  | 100 |  | 100 |
|  | Cefepime | ≤0.25 | 4 | ≤0.25 – >16 |  | 94.8 |  | 81.0 |
|  | Meropenem | 0.06 | 0.25 | 0.015 – 32 |  | 94.8 |  | 94.8 |
| *Enterobacter* *hormaechei* (163) | Cefepime-taniborbactam | 0.06 | 1 | 0.03 – 16 |  | 100 |  | 100 |
|  | Cefepime | ≤0.25 | >16 | ≤0.25 – >16 |  | 77.9 |  | 62.6 |
|  | Meropenem | 0.03 | 2 | 0.015 – 32 |  | 89.0 |  | 90.8 |
| *Enterobacter* sp. (unable to speciate) (179) | Cefepime-taniborbactam | 0.06 | 0.5 | ≤0.008 – 4 |  | 100 |  | 100 |
|  | Cefepime | ≤0.25 | 8 | ≤0.25 – >16 |  | 91.1 |  | 75.4 |
|  | Meropenem | 0.03 | 0.25 | 0.015 – 32 |  | 96.6 |  | 97.8 |
| *Escherichia* *coli* (5717) | Cefepime-taniborbactam | 0.03 | 0.12 | ≤0.008 – >64 |  | 99.7 |  | 99.7 |
|  | Cefepime | ≤0.25 | >16 | ≤0.25 – >16 |  | 83.2 |  | 74.2 |
|  | Meropenem | 0.03 | 0.06 | ≤0.004 – >64 |  | 98.8 |  | 98.9 |
| *Klebsiella* *aerogenes* (606) | Cefepime-taniborbactam | 0.06 | 0.12 | ≤0.008 – 4 |  | 100 |  | 100 |
|  | Cefepime | ≤0.25 | 1 | ≤0.25 – >16 |  | 97.0 |  | 92.4 |
|  | Meropenem | 0.06 | 0.12 | 0.015 – 32 |  | 97.2 |  | 98.2 |
| *Klebsiella* *oxytoca* (1094) | Cefepime-taniborbactam | 0.03 | 0.06 | ≤0.008 – 8 |  | 100 |  | 100 |
|  | Cefepime | ≤0.25 | 1 | ≤0.25 – >16 |  | 97.7 |  | 92.0 |
|  | Meropenem | 0.03 | 0.06 | ≤0.004 – 64 |  | 98.9 |  | 99.2 |
| *Klebsiella* *pneumoniae* (5700) | Cefepime-taniborbactam | 0.06 | 1 | ≤0.008 – >64 |  | 98.8 |  | 98.8 |
|  | Cefepime | ≤0.25 | >16 | ≤0.25 – >16 |  | 68.7 |  | 61.7 |
|  | Meropenem | 0.03 | 16 | ≤0.004 – >64 |  | 85.9 |  | 87.2 |
| *Morganella* *morganii* (634) | Cefepime-taniborbactam | 0.03 | 0.06 | ≤0.008 – 64 |  | 99.8 |  | 99.8 |
|  | Cefepime | ≤0.25 | ≤0.25 | ≤0.25 – >16 |  | 97.8 |  | 95.0 |
|  | Meropenem | 0.12 | 0.25 | ≤0.004 – >32 |  | 99.1 |  | 99.1 |
| *Proteus mirabilis* (1435) | Cefepime-taniborbactam | 0.06 | 0.12 | ≤0.008 – 8 |  | 100 |  | 100 |
|  | Cefepime | ≤0.25 | 4 | ≤0.25 – >16 |  | 91.6 |  | 84.9 |
|  | Meropenem | 0.06 | 0.12 | ≤0.004 – 64 |  | 98.6 |  | 98.7 |
| *Proteus vulgaris* (588) | Cefepime-taniborbactam | 0.06 | 0.06 | 0.015 – 8 |  | 100 |  | 100 |
|  | Cefepime | ≤0.25 | ≤0.25 | ≤0.25 – >16 |  | 99.7 |  | 98.3 |
|  | Meropenem | 0.06 | 0.12 | ≤0.004 – 16 |  | 99.7 |  | 99.7 |
| *Providencia rettgeri* (253) | Cefepime-taniborbactam | 0.015 | 0.12 | ≤0.008 – >64 |  | 96.4 |  | 96.4 |
|  | Cefepime | ≤0.25 | 0.5 | ≤0.25 – >16 |  | 94.1 |  | 92.1 |
|  | Meropenem | 0.06 | 0.12 | ≤0.004 – >64 |  | 94.9 |  | 94.9 |
| *Providencia stuartii (*335) | Cefepime-taniborbactam | 0.06 | 0.12 | 0.015 – 32 |  | 99.4 |  | 99.4 |
|  | Cefepime | ≤0.25 | 8 | ≤0.25 – >16 |  | 90.1 |  | 77.6 |
|  | Meropenem | 0.06 | 0.25 | 0.015 – >32 |  | 95.2 |  | 96.7 |
| *Serratia* *liquefaciens* (64) | Cefepime-taniborbactam | 0.06 | 0.12 | 0.015 – 0.5 |  | 100 |  | 100 |
|  | Cefepime | ≤0.25 | ≤0.25 | ≤0.25 – 0.5 |  | 100 |  | 100 |
|  | Meropenem | 0.06 | 0.06 | 0.015 – 0.12 |  | 100 |  | 100 |
| *Serratia* *marcescens* (757) | Cefepime-taniborbactam | 0.06 | 0.25 | 0.015 – >16 |  | 99.7 |  | 99.7 |
|  | Cefepime | ≤0.25 | 2 | ≤0.25 – >16 |  | 92.3 |  | 90.0 |
|  | Meropenem | 0.06 | 0.12 | ≤0.004 – >64 |  | 96.7 |  | 97.0 |
| *Serratia* sp. (unable to speciate) (280) | Cefepime-taniborbactam | 0.06 | 0.25 | ≤0.008 – 4 |  | 100 |  | 100 |
|  | Cefepime | ≤0.25 | 0.5 | ≤0.25 – >16 |  | 98.2 |  | 93.2 |
|  | Meropenem | 0.06 | 0.12 | 0.03 – 32 |  | 99.3 |  | 99.3 |

^a^ Species/groups listed were limited to those with 50 or more isolates tested.

^b^ For comparative purposes only, % susceptible values for cefepime-taniborbactam correspond to the percentage of isolates inhibited at ≤16 mg/L.

^c^ For cefepime: percent susceptible values by CLSI breakpoints include isolates with both susceptible (MIC ≤2 mg/L) and susceptible-dose dependent (MIC 4 and 8 mg/L) MICs

^d^ Isolates for which MALDI-TOF identified only to complex level.

**Table S7.** Cefepime-taniborbactam MIC distributions for 396 isolates of carbapenemase-positive *P. aeruginosa*^a^ stratified by carbapenemase type

|  | Cefepime-taniborbactam MIC, mg/L; number of isolates (cumulative % of isolates inhibited at MIC) | | | | | |  |
| --- | --- | --- | --- | --- | --- | --- | --- |
| Carbapenemase | ≤4 | 8 | 16 | 32 | ≥64 | Total | |
| VIM | 64 (23.6) | 129 (71.2) | 29 (81.9) | 10 (85.6) | 39 (100) | 271 | |
| GES^b^ | 11 (21.2) | 36 (90.4) | 5 (100) |  |  | 52 | |
| NDM^c^ |  | 1 (1.6) | 0 (1.6) | 18 (29.7) | 45 (100) | 64 | |
| KPC^d^ | 1 (11.1) | 3 (44.4) | 2 (66.7) | 1 (77.8) | 2 (100) | 9 | |
| Total (cumulative % inhibited^e^) | 76 (19.2) | 169 (61.9) | 36 (71.0) | 29 (78.3) | 86 (100) | 396 | |

^a^ IMP-positive isolates (*n*=67) were excluded from the dataset as IMP is outside the spectrum of taniborbactam inhibition; one isolate co-carrying VIM and NDM was also excluded.

^b^ Only GES variants with reported carbapenemase activity were included (i.e., GES-5, GES-6, and GES-20); GES-carriers co-carrying NDM or VIM were only included in the NDM and VIM groupings, respectively.

^c^ Includes two isolates co-carrying NDM and DIM (Dutch imipenemease).

^d^ KPC-positive isolates co-carrying VIM were only included in the VIM grouping.

^e^ Cumulative % of all carbapenemase-positive isolates inhibited at cefepime-taniborbactam MIC.

**Table S8.** *In vitro* activity of cefepime-taniborbactam, cefepime, and meropenem against 7919 clinical isolates of *P. aeruginosa* stratified by specimen source

|  |  | MIC, mg/L | | |  | MIC interpretation | | |
| --- | --- | --- | --- | --- | --- | --- | --- | --- |
|  | Antimicrobial agent | MIC_50_ | MIC_90_ | MIC range |  | CLSI % susceptible |  | EUCAST % susceptible |
| Specimen source  (no. of isolates; % of total isolates)^a^ |  |  |  |  |  |  |  |  |
| Blood (459; 5.8%) | Cefepime-taniborbactam^b^ | 2 | 8 | ≤0.06 – >128 |  | 96.9 |  | 96.9 |
|  | Cefepime^c^ | 4 | 32 | ≤0.25 – >32 |  | 78.6 |  | 78.6 |
|  | Meropenem | 0.5 | >8 | ≤0.06 – >64 |  | 73.2 |  | 73.2 |
| Intraabdominal (601; 7.6%) | Cefepime-taniborbactam | 2 | 8 | ≤0.06 – >128 |  | 97.2 |  | 97.2 |
|  | Cefepime | 4 | 32 | ≤0.25 – >32 |  | 81.7 |  | 81.7 |
|  | Meropenem | 0.5 | >8 | ≤0.06 – >64 |  | 72.9 |  | 72.9 |
| Respiratory (4983; 62.9%) | Cefepime-taniborbactam | 2 | 8 | ≤0.06 – >128 |  | 97.0 |  | 97.0 |
|  | Cefepime | 4 | 32 | ≤0.25 – >32 |  | 77.8 |  | 77.8 |
|  | Meropenem | 0.5 | >8 | ≤0.06 – >64 |  | 70.3 |  | 70.3 |
| Skin and soft tissue (641; 8.1%) | Cefepime-taniborbactam | 2 | 8 | ≤0.06 – >128 |  | 95.9 |  | 95.9 |
|  | Cefepime | 4 | 32 | ≤0.25 – >32 |  | 78.0 |  | 78.0 |
|  | Meropenem | 0.5 | >8 | ≤0.06 – >64 |  | 77.5 |  | 77.5 |
| Urine (1233; 15.6%) | Cefepime-taniborbactam | 2 | 8 | ≤0.06 – >128 |  | 94.2 |  | 94.2 |
|  | Cefepime | 4 | 32 | ≤0.25 – >32 |  | 79.6 |  | 79.6 |
|  | Meropenem | 0.5 | >8 | ≤0.06 – >64 |  | 74.9 |  | 74.9 |

^a^ Two *P. aeruginosa* isolates did not have a specimen source assigned.

^b^ For comparative purposes only, % susceptible values for cefepime-taniborbactam correspond to the percentage of isolates inhibited at ≤16 mg/L.

^c^ For cefepime tested against *P. aeruginosa* isolates, percent susceptible values by EUCAST breakpoints correspond to isolates with susceptible, increased exposure (MIC ≤8 mg/L) MICs.
